# Identification of C3H2C3-type RING E3 ubiquitin ligase in grapevine and characterization of drought resistance function of VyRCHC114

**DOI:** 10.1186/s12870-021-03162-8

**Published:** 2021-09-17

**Authors:** Yihe Yu, Shengdi Yang, Lu Bian, Keke Yu, Xiangxuan Meng, Guohai Zhang, Weirong Xu, Wenkong Yao, Dalong Guo

**Affiliations:** 1 College of Horticulture and Plant Protection, Henan University of Science and Technology, Luoyang, 471023 Henan Province China; 2 Henan Engineering Technology Research Center of Quality Regulation and Controlling of Horticultural Plants, Luoyang, 471023 Henan Province China; 3 School of Wine, Ningxia University, Yinchuan, 750021 Ningxia Province China

**Keywords:** RING, Drought stress, Grapevine, Ubiquitin, Overexpression

## Abstract

**Background:**

RING is one of the largest E3 ubiquitin ligase families and C3H2C3 type is the largest subfamily of RING, which plays an important role in plant growth and development, and growth and responses to biotic and abiotic stresses.

**Results:**

A total of 143 RING C3H2C3-type genes (*RCHCs*) were discovered from the grapevine genome and separated into groups (I-XI) according to their phylogenetic analysis, and these genes named according to their positions on chromosomes. Gene replication analysis showed that tandem duplications play a predominant role in the expansion of *VvRCHCs* family together. Structural analysis showed that most *VvRCHCs* (67.13 %) had no more than 2 introns, while genes clustered together based on phylogenetic trees had similar motifs and evolutionarily conserved structures. *Cis*-acting element analysis showed the diversity of *VvRCHCs* regulation. The expression profiles of eight DEGs in RNA-Seq after drought stress were like the results of qRT-PCR analysis. In vitro ubiquitin experiment showed that *VyRCHC114* had E3 ubiquitin ligase activity, overexpression of *VyRCHC114* in Arabidopsis improved drought tolerance. Moreover, the transgenic plant survival rate increased by 30 %, accompanied by electrolyte leakage, chlorophyll content and the activities of SOD, POD, APX and CAT were changed. The quantitative expression of *AtCOR15a*, *AtRD29A*, *AtERD15* and *AtP5CS1* showed that they participated in the response to drought stress may be regulated by the expression of *VyRCHC114*.

**Conclusions:**

This study provides valuable new information for the evolution of grapevine *RCHCs* and its relevance for studying the functional characteristics of grapevine *VyRCHC114* genes under drought stress.

**Supplementary Information:**

The online version contains supplementary material available at 10.1186/s12870-021-03162-8.

## Background

To survive in a changing environment, post-translational modification of proteins often occurs when plants perceive and transmit internal or external signals. The acetylation, methylation, phosphorylation, and ubiquitination of proteins are the main types of post-translational modification, which play a key role in different plant development stages and plant-environment interactions. The process of classifying intracellular proteins under the action of a variety of special enzymes, and specifically modifying the screened target proteins, is called ubiquitination [1]. In eukaryotic cells, the ubiquitin sfigystem is complex and mainly involving ubiquitin (a small molecule protein), intact 26 S proteasome, ubiquitin-activating enzyme (E1), ubiquitin-binding enzyme (E2), and ubiquitin-ligase (E3) [2]. The inactivated ubiquitin-dependent ATP is first activated by E1 through the thioester bond formed between the C-terminal of ubiquitin and the cysteine residue of E1; then the ubiquitin signal connected to E1 is transferred to the acetylcysteine of E2. In the next step, the ubiquitin linked to E2 is transferred directly or indirectly to the lysine residue of the target protein via E3. It is noteworthy that E3 ubiquitin ligase is the main factor determining the binding of specific protein during ubiquitination process [3], it can repeatedly add ubiquitin to the substrate protein, so that the target protein is degraded by the 26 S protease [4].

E3 ubiquitin ligases can be divided into 9 categories based on specific conserved domains: RING, HECT, U-box, F-box, cullin, BTB, DDB, RBX and SKP. RING E3 ligases protein has a conserved RING domain, which can provide residence sites for E2 and specific substrates and enable E2-bound ubiquitin molecules to transfer to the host protein, thus completing the ubiquitination process. In the RING domain, there are eight conserved amino acids (Cys or His) located in the center of the three-dimensional protein structure, which can combine with two zinc ions to help stabilize the entire structure. According to the types of conserved amino acid sites, they are divided into different subfamily. Among them, RING C3H2C3 is the largest subfamily. The RING conserved domain sequence of this family member is Cys-*X*2-Cys-*X*(9–39)-Cys-*X*(1–3)-His-*X*(2–3)-His-*X*2-Cys-*X*(4–48)-Cys-*X*2-Cys, *X* is any amino acid. In recent years, a growing number of studies have shown that the RING E3 ligase gene also figure prominently in abiotic stress responses of plants [5]. *SpRing* is a RING-type E3 ubiquitin ligase located in endoplasmic reticulum and participates in salt stress signal transmission in wild tomato variety *Solanum pimpinellifolium* ‘PI365967’. In addition, *SpRing* is silenced by virus-induced gene silencing, resulting in increased sensitivity of wild tomato to salt stress. Overexpression of *SpRing* in Arabidopsis can improve its salt tolerance [6]. SDIR1 (SALT AND DROUGHT-INDUCED REALLY INTERESTING NEW GENE FINGER1) is a RING-type E3 ubiquitin ligase that regulates the salt stress response and ABA signaling in Arabidopsis by degrading the target protein SDIRIP1 (SDIR1-INTERACTING PROTEIN1). The downstream transcription factor ABI5 (ABA-INSENSITIVE5) is regulated by SDIRIP1, and overexpression of ABI5 increases salt tolerance [7]. The E3 ubiquitin ligase *OsHTAS* (*Oryza sativa* HEAT TOLERANCE AT SEEDLING STAGE) regulates the stomatal opening state of leaves by regulating ROS homeostasis, thus improving the basal heat resistance of leaves. It involves two pathways, ABA-mediated [8]. In Arabidopsis, CHYR1 (CHYZINC-FINGERANDRINGPROTEIN1) encodes the RING-type E3 ubiquitin ligase which interacts with the related protein kinase KINASE2 (SnRK2) and can be phosphorylated by SnRK2.6 on its Thr-178 residues. When mediated by ABA, CHYR1 promotes the production of reactive oxygen species (ROS), stomatal closure, and drought tolerance in plants [9]. The capsicum annular E3 ubiquitin ligase, CaAIRF1 (*Capsicum annuum* ADIP1 INTERACTING RING FINGER PROTEIN1), can interact with protein phosphatase CaADIP1 and positively regulate ABA signaling pathway to improve drought tolerance [10]. In *Zea mays*, ZmXerico1 encodes a RING-type E3 ligase, which can regulate the stability of ABA8’-hydroxylase protein and thereby enable control of the dynamic balance of ABA, hence, expression of *ZmXerico1* endows maize plants with ABA sensitivity and improves their water use efficiency under drought stress [11]. Furthermore, Arabidopsis AtAIRP1, AtAIRP2, AtAIRP3 and CaAIR1 jointly encode an E3 ubiquitin ligase, which regulates drought responses by regulating ABA signaling transduction, the expression of these genes increases ABA-mediated stomatal closure [12–15]. Collectively, the above studies show that E3 ligase plays a crucial role in response to abiotic stress.

Grapevine (*Vitis vinifera* L.) is a major cash crop, whose cultivated varieties have a total worldwide output of nearly 70 million tons of the fruit berries from more 7 million hectares of harvested land [16]. This plant is mainly grown to produce table grapes, fruit juices, and wine [17]. Most grapevine producing areas in the world incur seasonal droughts. According to global climate modeling, droughts will intensify in the near future. Drought can adversely affect the growth and development of grapevines, because under drought stress the concentration of cytokinin in grape stems decreases, vegetative and reproductive growth are inhibited [18]. When grapevines are in full bloom, drought stress will also affect the pollination process, which decreases the fruit setting rate and affects the size of the individual fruit berries produced [19]. With worsening water shortages, drought stress is likely to become a key factor impacting grapevine and wine production worldwide [20]. Therefore, it is of great significance to grapevine production and breeding to study the drought resistance of wild grapevine plants as this could uncover the molecular mechanisms enabling them to withstand drought effects. *Vitis yeshanensis* is a wild grapevine plant native to arid areas of China, whose morphological characteristics indicate adaptability to arid environments in many aspects [21]. Several studies have shown that wild *Vitis yeshanensis* has stronger drought resistance than other cultivars [22, 23].

The RING-type gene family has been found in more and more plant species, and its importance in plant stress responses and growth and development has been recognized, but RING-type genes have not been fully identified in grapevine. It is reported that the RING type E3 ubiquitin ligase is involved in grapevine stress and growth, but few studies have investigated the involvement of E3 ubiquitin ligase in regulating grapevine response to drought stress. We assume that RCHC protein may mediate the ubiquitination of key factors during grape drought stress to regulate plant drought resistance. This study aimed to characterize the RING-type E3 ubiquitin ligase in grapevine’s genome and its relevance for drought stress. Genome-wide identification of C3H2C3 genes, the largest subfamily of grapevine RING-type, was carried out, coupled to their phylogenetic analysis, gene structure analysis, chromosome mapping, gene replication analysis, and *cis-*acting element analysis in gene promoter regions. We also quantified the expression levels of these genes under simulated drought treatment, 136 *RCHC* genes were found to be expressed and 52 DEGs, which 8 DEGs at least 3 stages. The *VyRCHC114* gene was confirmed by RT-qPCR, and then the ubiquitin ligase activity of the gene was verified. The function of the gene under drought conditions was elucidated using Arabidopsis transgenic plants. Our study provides an important basis for the involvement of RCHC protein in the regulation of grape ubiquitination under drought stress.

## Results

### Genome-wide identification of RING C3H2C3 type finger proteins in grapevine

The results of the Hidden Markov Model (HMM) were analyzed, and the gene sequences were extracted and given to SMART, CDD, and Pfam for domain authentication. From this, 143 *VvRCHC* genes were obtained by comparing and screening genes with eight conservative metal ligands, and the alignment members were not abandoned. The physicochemical properties of 143 *VvRCHCs* were identified (Table 1). The number of amino acids encoded by the 143 *VvRCHCs* ranged from 70 (*VvRCHC50*) to 763 (*VvRCHC98*). For these genes, the molecular weights of their products varied from 7.83 kDa to 83.58 kDa, while their isoelectric points varied from 3.88 to 9.95.

**Table 1:** Detailed information of all 143 VvRCHCs identified in grapevine genome

Gene name	Sequence ID	Chromosome	Localization	Protein Length (***aa***)^**a**^	Molecular Weight (kDa)^**b**^	***pI***^**c**^
*VvRCHC1*	VIT_01s0011g00090.t01	Chr1	116646~118695	167	18.65	4.9
*VvRCHC2*	VIT_01s0011g02350.t01	Chr1	2131084~2131795	174	20.07	8.32
*VvRCHC3*	VIT_01s0011g02360.t01	Chr1	2136955~2137455	166	18.87	7.05
*VvRCHC4*	VIT_01s0011g02380.t01	Chr1	2145560~2145874	104	12.09	5.06
*VvRCHC5*	VIT_01s0011g02390.t01	Chr1	2147401~2148062	172	19.91	5.88
*VvRCHC6*	VIT_01s0011g02410.t01	Chr1	2170994~2171522	173	19.97	7.57
*VvRCHC7*	VIT_01s0011g02420.t01	Chr1	2187275~2187979	173	19.70	6.64
*VvRCHC8*	VIT_01s0011g04080.t01	Chr1	3727811~3730410	351	38.67	9.46
*VvRCHC9*	VIT_01s0026g00300.t01	Chr1	8988327~8989738	420	45.76	6.01
*VvRCHC10*	VIT_01s0026g02540.t01	Chr1	12168327~12169146	233	25.68	4.87
*VvRCHC11*	VIT_01s0150g00260.t01	Chr1	22675690~22682439	232	25.76	5.25
*VvRCHC12*	VIT_02s0025g00030.t01	Chr2	209926~211750	227	24.95	4.4
*VvRCHC13*	VIT_02s0025g00140.t01	Chr2	291046~291759	168	19.26	4.66
*VvRCHC14*	VIT_02s0025g01430.t01	Chr2	1379376~1383064	386	42.37	6.09
*VvRCHC15*	VIT_02s0025g03070.t01	Chr2	2622704~2623554	187	20.18	5.72
*VvRCHC16*	VIT_02s0025g04150.t01	Chr2	3670653~3678402	384	42.55	5.23
*VvRCHC17*	VIT_02s0012g01440.t01	Chr2	7633461~7635180	292	33.14	4.96
*VvRCHC18*	VIT_02s0087g00420.t01	Chr2	17748161~17758528	561	62.51	8.15
*VvRCHC19*	VIT_03s0063g00160.t01	Chr3	3768331~3782384	734	78.34	6.06
*VvRCHC20*	VIT_03s0063g01890.t01	Chr3	5217040~5219107	396	45.16	9.95
*VvRCHC21*	VIT_03s0091g00480.t01	Chr3	6851584~6855075	278	31.25	8.63
*VvRCHC22*	VIT_03s0088g00930.t01	Chr3	9146189~9147403	393	42.59	6.53
*VvRCHC23*	VIT_03s0088g01090.t01	Chr3	9340156~9341562	457	49.83	6.71
*VvRCHC24*	VIT_03s0097g00680.t01	Chr3	11239802~11241097	421	45.90	6.41
*VvRCHC25*	VIT_03s0017g00670.t01	Chr3	15529867~15532640	427	47.50	5.43
*VvRCHC26*	VIT_04s0008g02290.t01	Chr4	1889354~1891807	293	32.81	5.02
*VvRCHC27*	VIT_04s0008g02390.t01	Chr4	1970602~1975816	550	60.01	8.19
*VvRCHC28*	VIT_04s0008g04280.t01	Chr4	3660980~3669030	401	44.19	7.88
*VvRCHC29*	VIT_04s0008g04480.t01	Chr4	3852636~3861794	315	36.68	7.19
*VvRCHC30*	VIT_04s0023g03460.t01	Chr4	20022053~20022628	166	18.05	6.97
*VvRCHC31*	VIT_05s0077g01970.t01	Chr5	1538565~1539938	317	34.99	8.34
*VvRCHC32*	VIT_05s0020g01800.t01	Chr5	3519576~3525991	267	31.02	6.58
*VvRCHC33*	VIT_05s0020g04000.t01	Chr5	5689012~5690195	164	18.50	9.8
*VvRCHC34*	VIT_05s0049g00480.t01	Chr5	7527982~7529676	390	43.91	8.25
*VvRCHC35*	VIT_05s0051g00730.t01	Chr5	11700888~11720325	190	21.26	6.14
*VvRCHC36*	VIT_06s0004g00120.t01	Chr6	257501~259353	368	40.90	4.82
*VvRCHC37*	VIT_06s0004g01930.t01	Chr6	2373671~2382922	252	28.69	5.47
*VvRCHC38*	VIT_06s0004g05080.t01	Chr6	6014664~6018771	284	31.83	5.73
*VvRCHC39*	VIT_06s0004g05090.t01	Chr6	6020330~6022057	386	42.57	8.87
*VvRCHC40*	VIT_06s0004g06930.t01	Chr6	7643002~7643805	267	29.33	5.76
*VvRCHC41*	VIT_06s0004g08080.t01	Chr6	8843648~8845413	263	29.12	7.89
*VvRCHC42*	VIT_06s0009g02350.t01	Chr6	14742953~14747814	336	36.99	6.3
*VvRCHC43*	VIT_06s0009g03540.t01	Chr6	16816009~16816598	162	18.43	5.32
*VvRCHC44*	VIT_06s0061g00710.t01	Chr6	18243947~18247168	289	33.05	6.45
*VvRCHC45*	VIT_07s0104g01370.t01	Chr7	2388448~2393119	268	31.42	6.1
*VvRCHC46*	VIT_07s0005g00710.t01	Chr7	3351600~3352612	263	28.82	6.7
*VvRCHC47*	VIT_07s0005g03120.t01	Chr7	5941893~5942809	264	29.97	6.11
*VvRCHC48*	VIT_07s0191g00230.t01	Chr7	15035569~15037412	372	41.30	5.98
*VvRCHC49*	VIT_07s0031g00370.t01	Chr7	16601231~16602190	145	16.55	4.66
*VvRCHC50*	VIT_07s0031g00380.t01	Chr7	16612579~16612919	70	7.83	4.93
*VvRCHC51*	VIT_07s0031g00390.t01	Chr7	16615612~16618016	244	26.72	4.48
*VvRCHC52*	VIT_07s0031g00400.t01	Chr7	16623222~16623766	102	11.17	4.45
*VvRCHC53*	VIT_07s0031g00440.t01	Chr7	16651426~16652337	220	24.25	4.17
*VvRCHC54*	VIT_07s0031g01270.t01	Chr7	17354201~17354899	159	18.65	5.95
*VvRCHC55*	VIT_07s0031g02250.t01	Chr7	18383520~18384391	182	19.29	8.32
*VvRCHC56*	VIT_08s0056g00320.t01	Chr8	453669~461038	590	66.55	4.63
*VvRCHC57*	VIT_08s0058g01270.t01	Chr8	10736680~10737686	195	21.19	6.91
*VvRCHC58*	VIT_08s0040g00310.t01	Chr8	11231370~11255659	385	43.10	6.81
*VvRCHC59*	VIT_08s0040g00590.t01	Chr8	11540810~11542129	314	34.85	7.7
*VvRCHC60*	VIT_08s0040g02160.t01	Chr8	13268900~13272455	546	60.42	6.26
*VvRCHC61*	VIT_08s0040g02950.t01	Chr8	13968494~13969465	285	31.37	7.6
*VvRCHC62*	VIT_08s0007g00150.t01	Chr8	14536064~14536944	178	19.87	5.78
*VvRCHC63*	VIT_08s0007g00720.t01	Chr8	14916670~14922764	516	57.58	8.62
*VvRCHC64*	VIT_08s0007g04790.t01	Chr8	18767623~18771322	289	33.33	6.11
*VvRCHC65*	VIT_09s0002g00220.t01	Chr9	197510~198755	304	33.08	5.68
*VvRCHC66*	VIT_09s0002g01500.t01	Chr9	1282580~1283385	140	15.83	4.92
*VvRCHC67*	VIT_09s0002g05120.t01	Chr9	4813804~4820015	442	48.81	6.5
*VvRCHC68*	VIT_09s0002g05130.t01	Chr9	4821806~4822755	208	22.69	5.82
*VvRCHC69*	VIT_09s0002g05140.t01	Chr9	4836806~4838491	309	33.91	5.16
*VvRCHC70*	VIT_10s0003g00850.t01	Chr10	2100756~2101775	218	23.74	5.19
*VvRCHC71*	VIT_10s0042g00580.t01	Chr10	13534118~13544150	308	33.77	3.88
*VvRCHC72*	VIT_11s0016g00070.t01	Chr11	37814~38430	136	15.38	8.71
*VvRCHC73*	VIT_11s0016g01430.t01	Chr11	1146136~1146786	178	20.16	6.38
*VvRCHC74*	VIT_11s0016g03190.t01	Chr11	2563204~2564128	168	17.85	6.39
*VvRCHC75*	VIT_11s0016g03420.t01	Chr11	2778391~2787457	469	53.37	7.24
*VvRCHC76*	VIT_11s0016g04450.t01	Chr11	3757897~3762458	407	44.19	5.53
*VvRCHC77*	VIT_11s0118g00640.t01	Chr11	6397984~6398548	119	13.64	4.55
*VvRCHC78*	VIT_11s0118g00760.t01	Chr11	6533770~6542103	542	58.69	5.35
*VvRCHC79*	VIT_11s0118g00780.t01	Chr11	6552717~6553829	193	21.59	9.37
*VvRCHC80*	VIT_11s0037g01400.t01	Chr11	10944676~10946544	543	59.50	6.26
*VvRCHC81*	VIT_11s0065g01210.t01	Chr11	15329284~15329979	167	18.78	8.82
*VvRCHC82*	VIT_11s0052g00360.t01	Chr11	17683751~17684146	131	14.89	4.83
*VvRCHC83*	VIT_11s0052g00530.t01	Chr11	17936593~17938168	457	51.48	9.56
*VvRCHC84*	VIT_12s0028g01220.t01	Chr12	1843979~1851603	219	24.66	4.88
*VvRCHC85*	VIT_12s0028g01560.t01	Chr12	2265770~2266849	190	22.29	8.77
*VvRCHC86*	VIT_12s0028g01570.t01	Chr12	2274491~2275931	224	26.20	7.62
*VvRCHC87*	VIT_12s0028g01580.t01	Chr12	2278915~2280538	339	38.82	6.63
*VvRCHC88*	VIT_12s0028g02530.t01	Chr12	3292336~3293351	254	27.54	5.72
*VvRCHC89*	VIT_12s0028g03410.t01	Chr12	4186053~4209365	570	65.51	8.3
*VvRCHC90*	VIT_12s0057g01330.t01	Chr12	10069923~10070893	202	22.49	5.85
*VvRCHC91*	VIT_12s0034g01390.t01	Chr12	17398801~17400234	451	52.26	8.22
*VvRCHC92*	VIT_12s0034g01400.t01	Chr12	17414404~17415189	261	29.71	6.86
*VvRCHC93*	VIT_13s0067g02880.t01	Chr13	1558971~1559978	197	21.06	6.73
*VvRCHC94*	VIT_13s0019g00990.t01	Chr13	2740552~2741055	167	18.34	5.94
*VvRCHC95*	VIT_13s0019g01000.t01	Chr13	2742040~2742492	150	16.69	4.64
*VvRCHC96*	VIT_13s0019g01020.t01	Chr13	2749369~2749848	159	17.49	6.77
*VvRCHC97*	VIT_13s0019g01960.t01	Chr13	3261036~3269087	275	30.08	6.23
*VvRCHC98*	VIT_13s0019g01980.t01	Chr13	3282264~3294013	763	83.58	7.79
*VvRCHC99*	VIT_13s0019g04100.t01	Chr13	5376959~5389168	565	63.18	6.42
*VvRCHC100*	VIT_13s0074g00370.t01	Chr13	7973641~7981844	209	23.54	5.82
*VvRCHC101*	VIT_13s0084g00140.t01	Chr13	18797445~18798197	191	20.08	7.57
*VvRCHC102*	VIT_13s0064g01030.t01	Chr13	22907333~22911899	247	28.32	5.45
*VvRCHC103*	VIT_13s0156g00140.t01	Chr13	23884150~23887948	312	33.41	4.7
*VvRCHC104*	VIT_14s0060g00290.t01	Chr14	278763~286055	137	16.21	7.58
*VvRCHC105*	VIT_14s0128g00120.t01	Chr14	2814506~2816124	420	45.76	9.5
*VvRCHC106*	VIT_14s0083g00710.t01	Chr14	22855118~22856402	391	43.71	3.97
*VvRCHC107*	VIT_14s0083g01000.t01	Chr14	23286167~23293023	225	25.54	4.94
*VvRCHC108*	VIT_14s0066g01610.t01	Chr14	27970245~27971983	386	42.89	6.16
*VvRCHC109*	VIT_15s0024g01990.t01	Chr15	4356512~4356925	137	14.73	5.93
*VvRCHC110*	VIT_15s0045g00330.t01	Chr15	5087898~5088931	338	37.58	5.55
*VvRCHC111*	VIT_15s0021g00890.t01	Chr15	10761195~10763021	203	22.12	4.78
*VvRCHC112*	VIT_15s0048g01840.t01	Chr15	15988642~15991207	201	22.58	5.51
*VvRCHC113*	VIT_15s0048g02030.t01	Chr15	16165343~16170176	382	43.09	5.65
*VvRCHC114*	VIT_15s0046g00930.t01	Chr15	17988199~17990082	372	40.71	4.78
*VvRCHC115*	VIT_15s0046g01820.t01	Chr15	18650570~18653922	184	20.52	4.66
*VvRCHC116*	VIT_15s0046g01880.t01	Chr15	18703282~18703952	167	19.10	4.83
*VvRCHC117*	VIT_15s0046g02070.t01	Chr15	18873629~18874670	197	21.47	6.49
*VvRCHC118*	VIT_15s0046g03770.t01	Chr15	20300677~20302386	81	9.47	6.47
*VvRCHC119*	VIT_16s0039g02200.t01	Chr16	2285896~2293114	502	56.87	6.57
*VvRCHC120*	VIT_16s0022g00290.t01	Chr16	11163433~11164324	76	8.64	7.57
*VvRCHC121*	VIT_16s0022g00600.t01	Chr16	11897657~11912487	587	65.27	6.85
*VvRCHC122*	VIT_16s0098g00250.t01	Chr16	20642143~20644650	367	40.74	5.45
*VvRCHC123*	VIT_17s0000g03210.t01	Chr17	3094227~3100434	371	40.80	8.7
*VvRCHC124*	VIT_17s0000g04730.t01	Chr17	5126163~5127854	439	49.33	4.13
*VvRCHC125*	VIT_17s0000g06460.t01	Chr17	7045832~7050297	217	49.33	4.13
*VvRCHC126*	VIT_17s0053g00320.t01	Chr17	14868182~14869346	369	38.60	4.43
*VvRCHC127*	VIT_18s0122g00870.t01	Chr18	626783~627577	184	19.24	8.28
*VvRCHC128*	VIT_18s0001g01050.t01	Chr18	1721216~1721614	132	14.79	5.02
*VvRCHC129*	VIT_18s0001g01060.t01	Chr18	1727361~1728295	114	12.33	4.84
*VvRCHC130*	VIT_18s0001g02280.t01	Chr18	2629486~2630245	207	23.33	3.99
*VvRCHC131*	VIT_18s0001g03270.t01	Chr18	3233803~3234804	333	38.18	7.13
*VvRCHC132*	VIT_18s0001g06640.t01	Chr18	5000284~5003505	405	44.98	5.15
*VvRCHC133*	VIT_18s0001g06670.t01	Chr18	5020431~5021751	398	44.25	9.09
*VvRCHC134*	VIT_18s0001g10260.t01	Chr18	8598100~8604904	734	78.55	5.98
*VvRCHC135*	VIT_18s0001g14530.t01	Chr18	12533609~12536412	334	37.20	4.34
*VvRCHC136*	VIT_18s0075g00220.t01	Chr18	21471547~21496753	444	47.91	8.92
*VvRCHC137*	VIT_18s0089g00860.t01	Chr18	28686085~28689586	221	24.97	5.05
*VvRCHC138*	VIT_19s0014g01850.t01	Chr19	2035536~2041782	538	58.57	6.3
*VvRCHC139*	VIT_19s0090g00400.t01	Chr19	6551526~6553119	220	24.03	6.51
*VvRCHC140*	VIT_19s0015g01000.t01	Chr19	9081946~9084990	343	39.31	8.81
*VvRCHC141*	VIT_00s0125g00250.t01	Un	1842078~1866688	497	56.09	5.07
*VvRCHC142*	VIT_00s0264g00020.t01	Un	18991085~18993638	235	25.36	5.02
*VvRCHC143*	VIT_00s0349g00040.t01	Un	24940977~24941538	135	15.22	8.12

^a^*aa* amino acid

^b^*kDa* kilo Dalton

^c^*pI* isoelectric point

### Analysis of *VvRCHCs* in the C3H2C3 domain

**Fig. 1:**
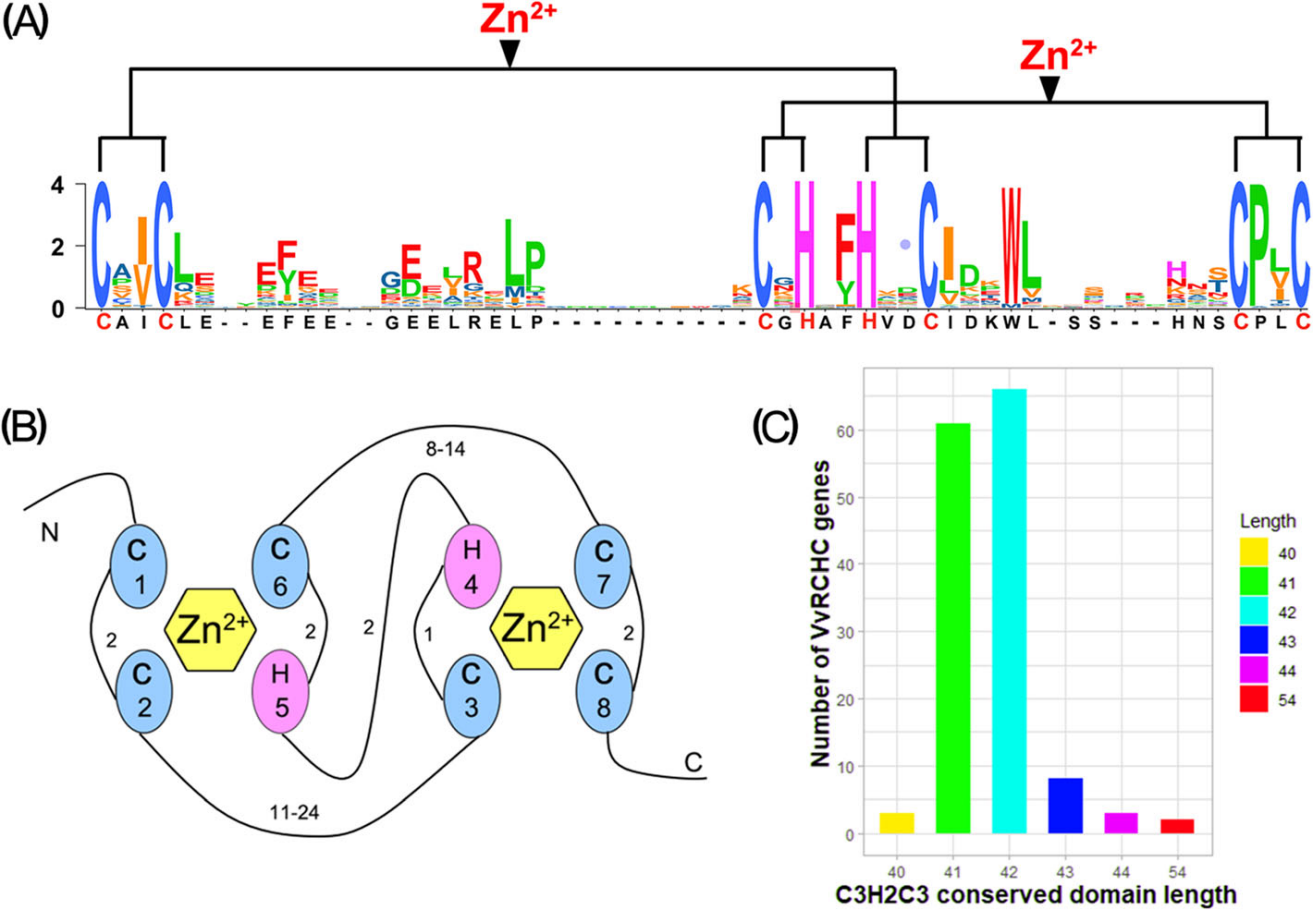
*VvRCHCs* C3H2H3 domain analysis. **a** Sequence of C3H2H3 domain. **b** Schematic diagram of zinc ion binding to the C3H2C3 structure of the *VvRCHCs*. Cysteine and histidine are metal ligands marked numerically in ellipses. The hexagon represents a zinc ion. The number on the line between metal ligands is the number of amino acids between them. **c** The number of different amino acids in the *VvRCHCs* in the metal ligand region

The typical RING domain is considered to be an octahedral group of metal-bound cysteine and its residues, which can chelate two zinc ions in a spherical cross-supported structure, in which the metal ligands 1 and 3, 2 and 4, each bind to one zinc ion. This structure requires a certain distance between adjacent metal ligands, it being variable between ml2 ~ ml3 and ml6 ~ ml7. We calculated statistics for this distance between adjacent metal ligands (Table S2). It was found that, except those between ml2 ~ ml3 and ml6 ~ ml7, the distances between other metal ligands were constant, while those from ml2 to ml3 spanned 11 to 24 amino acids, and for ml6 ~ ml7 the distance varied from 8 to 14 amino acids. The 143 *VvRCHCs* C3H2C3 domains have two amino acids between ml1 ~ ml2 and ml5 ~ ml6, while ml3 ~ ml4 contains one amino acid, ml7 ~ ml8 contains two amino acids as does ml4 ~ ml5. To understand whether these RING C3H2C3 structural domains are conserved apart from their eight special metal ligands, their comparative analysis was conducted (Fig. S1). This revealed that some amino acids in the structural domain of RING C3H2C3 have a typical position bias (Fig. 1a). In the C3H2C3 type RING region, the ml2 located in front of amino acid residues is the most common Ile (I) or Val (V); likewise, the phenylalanine (Phe, F) residue is typically before ml5, the leucine residue (Leu, L) is always next to ml2, and the aspartic acid (Asp, D) residue is usually positioned after ml6, while the tryptophan residue (Trp, W) is usually the fourth following ml6. Notably, a very conservative proline (P) was found situated after ml7. According to the RING-type C3H2H3 domain schematic diagram, two pairs of metal ligands bind to a zinc ion (Fig. 1b). The total amino acid length of the C3H2C3 domain per *VvRCHC* gene and the corresponding number of different lengths were calculated: the vast majority of these were 41 and 42, accounting for 88.8 % of all genes (Fig. 1c).

### Phylogenetic analysis of *VvRCHCs*

**Fig. 2:**
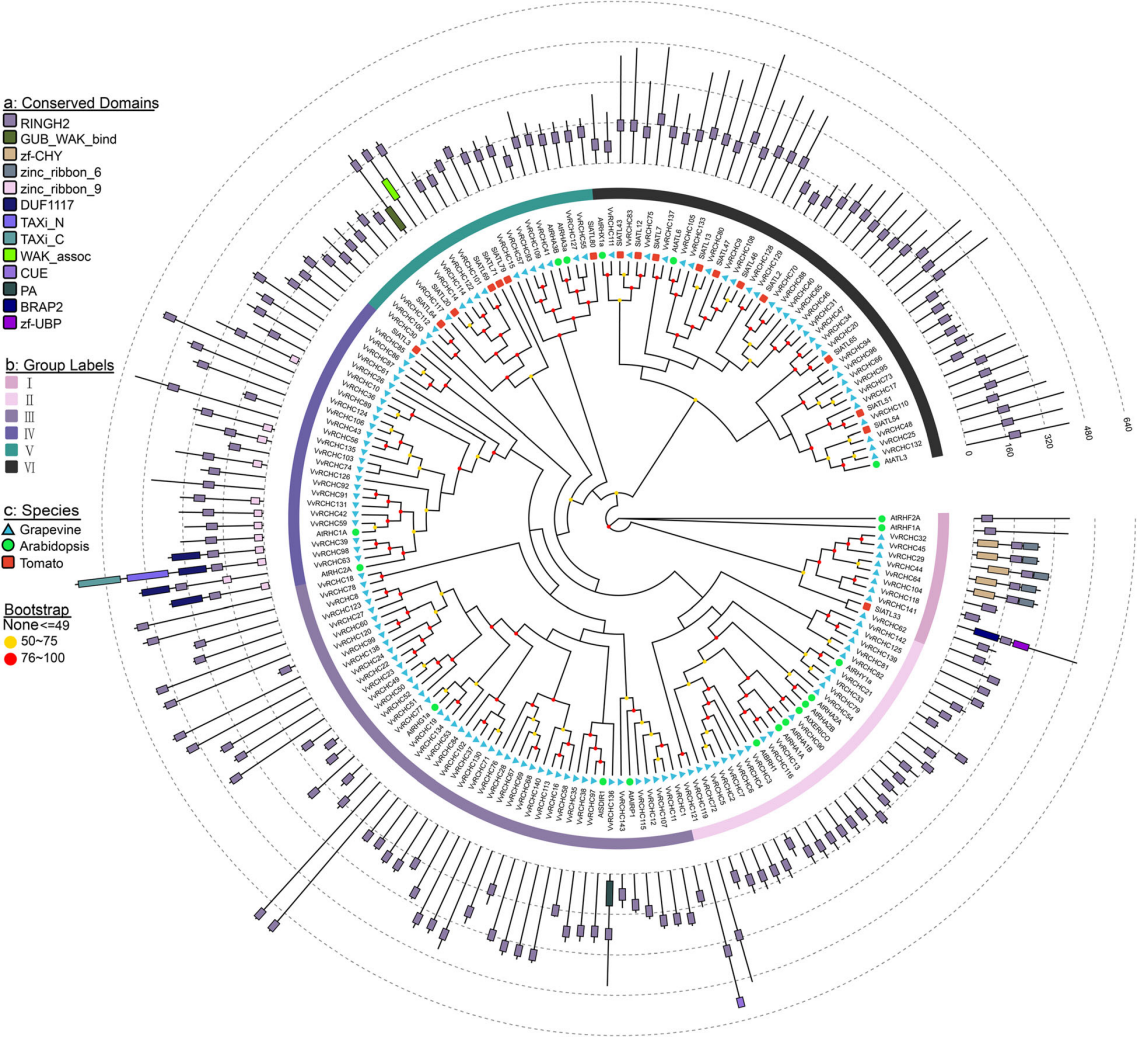
Phylogenetic analysis of RCHC protein in grapevine and other plants. Via the ML method, MEGA7.0 was used to construct phylogenetic trees of grapevine, Arabidopsis, and tomato (*Solanum lycopersicum*) with RING-type C3H2H3 proteins. The number of bootstrap repeats was n = 1000. Displayed are the percentages of bootstrap scores greater than 50 %. Conservative domains, grouping, and information on different species are shown in the form of **a**, **b**, **c**

**Fig. 3:**
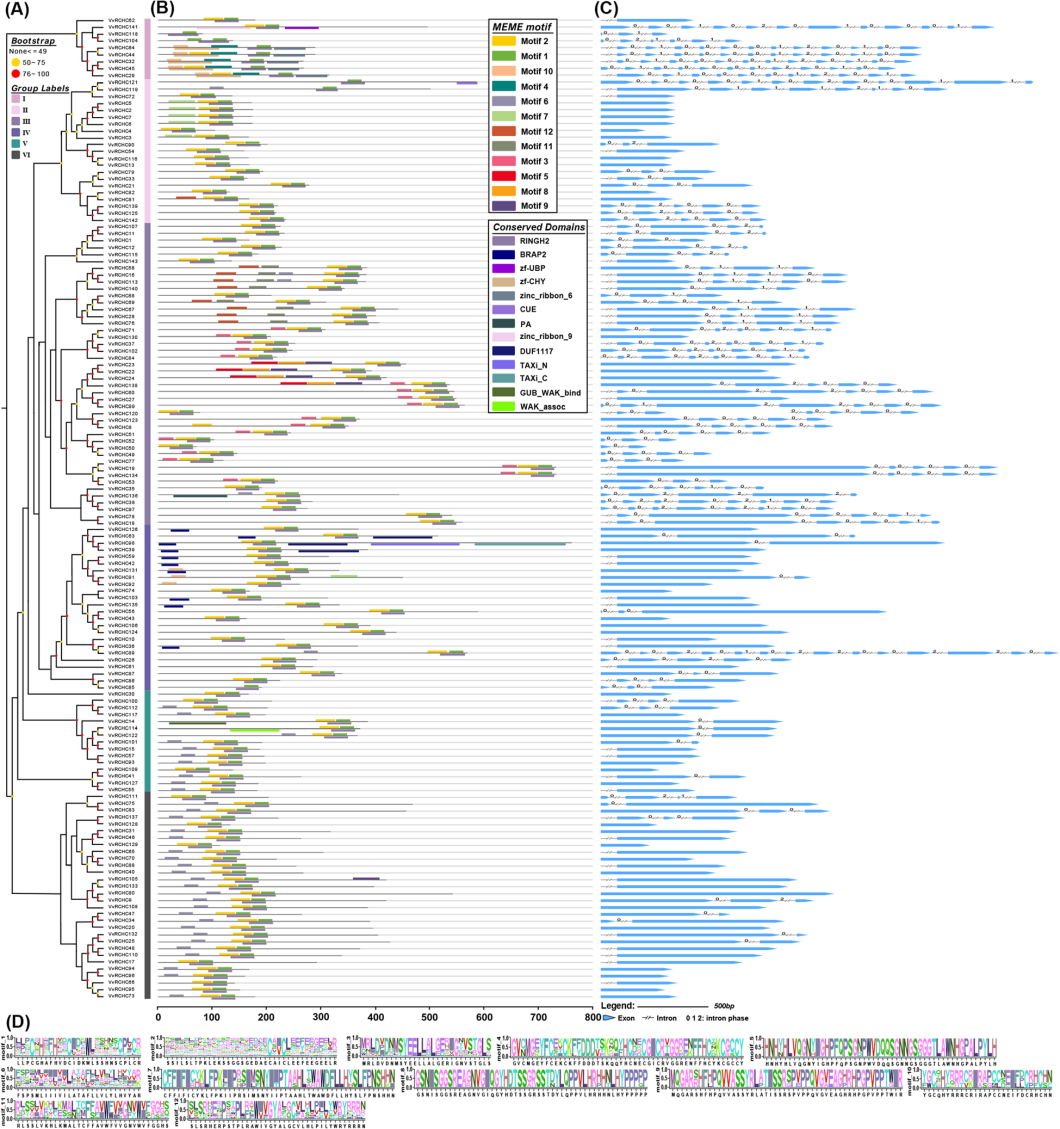
Phylogenetic tree, gene domain, and structure analysis of *VvRCHCs* in grapevine. **a** The phylogenetic tree of VvRCHCs was constructed using the ML method. Different background colors represent different grouping branches. **b** Domain analysis of VvRCHCs proteins. At the bottom of the line, different colored squares represent different types of conserved amino acid sequences and based on MEME analysis. The modules of different colors above the line represent the functional domains that have been identified. **c** Genetic structure of *VvRCHCs*, the CDS sequence is represented by a blue square/rectangle, the introns by black lines

To infer the evolutionary relationships of grapevine *VvRCHCs*, phylogenetic analysis of RCHC protein sequences of Arabidopsis, tomato, and grapevine were constructed (using the Maximum Likelihood method). According to the phylogenetic analysis, these 180 genes can be divided into 6 subgroups: I ~ VI (Fig. 2). Group I has the least number of members, only 12, and the group of the largest number of members is group III, while the *RCHC* gene of *Arabidopsis thaliana* or tomato is found in each group. It is worth noting that more *RCHC* genes of *Arabidopsis thaliana* and tomato are gathered in group VI. Most of the RING-type C3H2C3 genes of grapevine display some homology to *RCHC* genes of Arabidopsis or tomato. In addition, some gene pairs showed high similarity in different groups, which were confirmed in the distance of evolutionary relationship, the location of RING conserved domain and the length of protein sequence. For instance, *SlATL33* and *VvRCHC62*, *SlATL46* and *VvRCHC108*, *SlATL51* and *VvRCHC110*, *AtBRH1* and *VvRCHC116*, *AtRHA1A* and *VvRCHC13*, *AtSDIR1* and *VvRCHC97*, *AtRHC1A* and *VvRCHC59* etc. Next, a phylogenetic tree containing only 143 VvRCHC protein sequences was constructed. To facilitate their study and analysis, the 143 members were divided into 6 groups (I ~ VI) according to the classification and phylogenetic analysis of Fig. 3a, from which 27 pairs of genes with high homology were found. Based on their color-coded names, the VvRCHCs were then divided into six groups according to the number of conserved amino acids in their protein sequence.

### Characterization of the motifs and gene structure of *VvRCHCs*

To further understand the diversity in motif composition between *VvRCHCs*, the MEME analysis of VvRCHC proteins from groups I to VI was carried out. From this, 12 conserved motifs were identified in the VvRCHC protein, named motif 1 to motif 12 (Fig. 3b), in which motif 1 and motif 2 is found in almost every *VvRCHCs*, this motif combines to form the eight most important metal ligand (Cys-Cys-Cys-His-His-Cys-Cys-Cys) structures of every *VvRCHC* gene. Importantly, there are 13 such structures in some genes, such as PA, CUE, DUF1117, zinc_ribbon_9, and zf-CHY, among others. These structures domain could be relevant for the function of *VvRCHCs*. The sequence information of motif 1 ~ 12 is presented in Table 2; Fig. 3d (motif data). We next analyzed the exons, introns, and several key structures of *VvRCHCs* (Fig. 3c). Most *VvRCHCs* (67.13 %) had no more than 2 introns, with a maximum of 19 introns in *VvRCHC29* and none intron in 57 *VvRCHCs* (Fig. S3). The longest intron length was found in *VvRCHC141*.

**Table 2:** Motif data information in MEME analysis of *VvRCHCs*

Motifs	Protein sequences	Width
Motif1	LLPCGHAFHVDCIDKWLSSHNSCPLCR	width=27
Motif2	SSILSLTPKLEKSSGGSGEDAECAICLEEFEEGEELR	width=37
Motif3	MRLDVDNMSYEELLALGERIGNVSTGLS	width=28
Motif4	GVCMGEYFCEKCKFFDDDTSKQQYHCDECGICRVGGRENFFHCKKCGCCY	width=50
Motif5	HNHYHLVQGNYIGHPFQPSGNPWVDQQSGNNGSGGGTLAWNHGPALPYLH	width=50
Motif6	FSPIMLIIJVILATAFLLVLLLHVYAR	width=27
Motif7	CFFIFICHKLFPKIIPRSIMSNYIIPTAAHLTWAWDFLLHYSLFPNSHHN	width=50
Motif8	GSNISGGSREAGNVGIQGYHDTSSGRSSTDYLQPPVLHRHHNLHYPPPPP	width=50
Motif9	MQGARRHFHPQVVASSYRLATISSRSPVPPQVGVEAGHRHPGPVPPTWIR	width=50
Motif10	YGCQHYRRRCRIRAPCCNEIFDCRHCHN	width=28
Motif11	RLSSLVKHLKMALDCFFAVWFVIGNVWIFGGGS	width=33
Motif12	SLSRHERPSAPLFAWIVGYALGCVLTLPILYWRYRRRN	width=38

According to the phylogenetic analysis of *VvRCHCs* (Fig. 3a), 45 pairs of genes can be found in the evolutionary tree. The results of the MEME and gene structure analyses of these gene pairs were also similar (Fig. 3b and c). For example, the conserved motifs in the protein sequences of *VvRCHC44/64* are highly similar, and the structure type and length are also similar, such as for *VvRCHC94/96, VvRCHC38/97, VvRCHC18/78, VvRCHC28/67* and *VvRCHC11/107*, to name a few. Unexpectedly, the MEME analysis of *VvRCHC55/127, VvRCHC105/133*, and *VvRCHC13/116* gene pairs gave near identical results to those from the gene structure analysis, revealing a remarkably similar protein sequence length, gene structure length and the intron number among them. We thus speculate these four gene pairs may perform similar functions in grapevine plants.

### Chromosomal localization and gene replication analysis of *VvRCHCs*

**Fig. 4:**
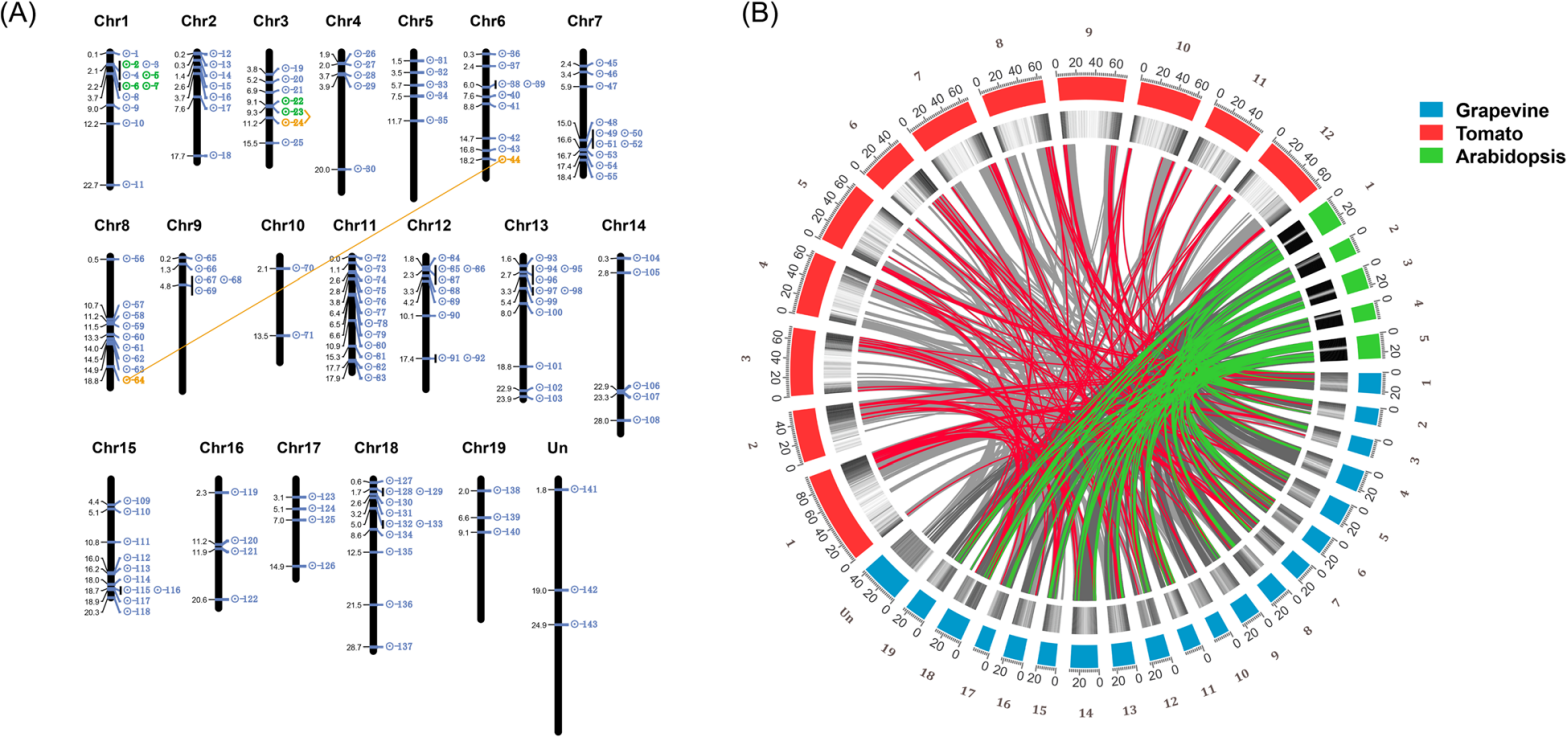
Chromosome location, gene replication, and collinear correlation. **a** Location information of *VvRCHCs* on grapevine chromosomes. Colored boxes and line connections represent the segmental repetitive gene pairs. **b** Collinear correlations of *RCHCs* in grapevine, tomato, and Arabidopsis. Each colored square is a chromosome (serial number). The black-and-white square shows the density of genes in each chromosome. Each line connects homologous genes; colored lines correspond to *RCHCs*, while gray lines denote other genes

According to the location of *VvRCHCs* in the grapevine genome, 143 *VvRCHCs* were placed on 20 chromosomes (Fig. 4a), albeit unevenly distributed among them. Imprinting of the *VvRCHCs* was found in each chromosome of grapevine, but the number of genes on different chromosomes varied. The most found were 12 *VvRCHCs* on chromosome 11, the 11 *VvRCHCs* were identified on chromosome 1,7,13 and 18. Further, we also observed that these most of these *VvRCHCs* are likely distributed at both ends of the chromosome, leaving only a small portion of them in its middle part. Gene replication events include tandem replication and segmental replication, which are very vital for expanding the number of members of the gene family. To clarify the amplification mechanism of *VvRCHCs* during their evolution, we studied their potential repetitive events of *VvRCHCs*. According to the intraspecific alignment of 143 *VvRCHCs*, 9 pairs of genes, 7 and 2, were respectively identified as associated with tandem or segmental replication events. Among the 9 pairs of gene events, the tandem repeat frequency between chromosomes 1 was the highest, there were six tandem replication events, moreover, one pair of genes on chromosomes 3 identified as tandem replication genes. These results suggested that the main replication event mode of grapevine *VvRCHCs* family is via tandem replication; hence, it could have played a crucial role in the amplification of *VvRCHCs* during their evolutionary history.

To explore the selection of grapevine *VvRCHCs* in terms of their repetition and differentiation, the non-synonymous (Ka), synonymous (Ks), and Ka/Ks of each duplicated *VvRCHCs* were calculated. Among the 9 pairs of repetitive genes in grapevine, the Ka/Ks values of one pair were less than 0.5, while the average Ka/Ks value was 0.325. It is worth noting that 8 pairs had Ka/Ks values less than 0.5, indicating that most of the repeated grapevine *VvRCHCs* were under negative selection during evolution (Table 3). Figure 4b shows that grapevine, Arabidopsis, and tomato all retained similar *RCHC* genes in their evolutionary history. It is worth noting the absence of homologous genes with *VvRCHC29* in tomato, but their presence in Arabidopsis, which may have arisen from gene deletions in the process of evolution, given that the same genes are *VvRCHC11*, *VvRCHC38*, *VvRCHC107*, *VvRCHC119*, and *VvRCHC137*. Nonetheless, two or more *RCHC* genes in Arabidopsis and tomato were found homologous to one *VvRCHC* gene; for example, *VvRCHC89* and *Solyc07g053850.3/Solyc12g005470.2* and *AT4G28370/AT2G20650*, as well as those of *VvRCHC1*, *VvRCHC32*, *VvRCHC97*, *VvRCHC104*, *VvRCHC118*, and *VvRCHC142*. Hence, these genes may be parallel gene pairs and the putative source of amplifications of *RCHC* genes during evolution.

**Table 3:** Ka/Ks analysis and divergence time estimated for grapvine duplicated VvRCHCs paralogs

Paralogous Pairs		Ka	Ks	Ka/Ks	Duplicate Type
*VvRCHC5*	*VvRCHC7*	0.143	0.674	0.212	tandem
*VvRCHC6*	*VvRCHC5*	0.146	0.341	0.429	tandem
*VvRCHC6*	*VvRCHC2*	0.093	0.191	0.486	tandem
*VvRCHC7*	*VvRCHC6*	0.080	0.277	0.290	tandem
*VvRCHC7*	*VvRCHC2*	0.106	0.285	0.372	tandem
*VvRCHC5*	*VvRCHC2*	0.226	1.102	0.205	tandem
*VvRCHC23*	*VvRCHC22*	0.017	0.040	0.431	tandem
*VvRCHC24*	*VvRCHC23*	0.019	0.020	0.951	segmental
*VvRCHC64*	*VvRCHC44*	0.166	0.929	0.178	segmental

### *Cis*-acting element analysis in *VvRCHCs* promoter

To further investigate the transcriptional regulation of *VvRCHCs*, *cis*-acting elements in the 2000 bp region upstream of the *VvRCHCs*’ codon was predicted. The predicted *cis-*acting elements can be divided into seven categories according to their functions: namely, light response (32), hormone response (11), growth and development response (9), stress response (6), enhanced promoter *cis*-acting (6), binding site *cis*-acting (6), and other functional *cis*-acting (2) elements. Most promoters of grapevine *VvRCHCs* contained the CAAT-box or TATA-box, which are involved in the enhanced promoter *cis*-acting elements. In addition, 127 *VvRCHCs* promoters harbored the stress response element ARE, more than half of the promoters of the *VvRCHCs* having the hormone response elements ABRE, TGACG-motif, CGTCA-motif, and over half of the *VvRCHCs* also featured the G-box, GT1-motif, and Box 4 in their promoters (Table S3). In the 2000 kb region upstream of *VvRCHCs*, discovered many different functions of cis element, in addition to the common cis element with light response and enhanced the promoter, also found that the more growth and adversity stress related cis element, this suggests that *VvRCHCs* may be widely participating in various life activities of plant.

It is known that the RING gene play a key role in plant growth and response to abiotic stresses. Accordingly, the *cis-*acting elements related to abiotic stress, growth and hormone regulation were focused upon here. The respective locations of the five major acting elements associated with hormone response, binding sites, growth and development, and stress of our concern, on the promoter of the *VvRCHCs* (Fig. S4a) were determined. To accurately identify the stress-related elements, we focused on four kinds (anaerobic induction, injury response, low temperature, drought response (Fig. S4b), low temperature response, defense and stress response), whose locations are also depicted. In addition, we counted the number of major elements related to stress, growth and development, and hormone responses in the *VvRCHC* gene promoter (Fig. S4b). Evidently, concerning growth and development, the number of O_2_-sites is the largest, there are 5 promoters of *VvRCHC6* and 4 promoters of *VvRCHC40*. In terms of stress, the number of ARE is very large, found in 89 % of the *VvRCHCs* promoters, moreover, 5 of the most promoters of *VvRCHC14* and *VvRCHC81* occurred. In terms of hormone response, the number of ABRE is dominant, found in 64 % of the *VvRCHCs* promoters, moreover, 9 of the most promoters of *VvRCHC3* and *VvRCHC16* occurred. Surprisingly, 22 of the *VvRCHC74* gene promoters were found and 11 of the *VvRCHC128* gene promoters were found. These results suggest that *VvRCHCs* may be associated with *cis-*acting elements of different functions; in other words, these genes may be regulated by these elements and thereby influence related plant life activities.

### Expression analysis of *VvRCHCs* in roots of two grapevine rootstocks with different drought sensitivity

**Fig. 5:**
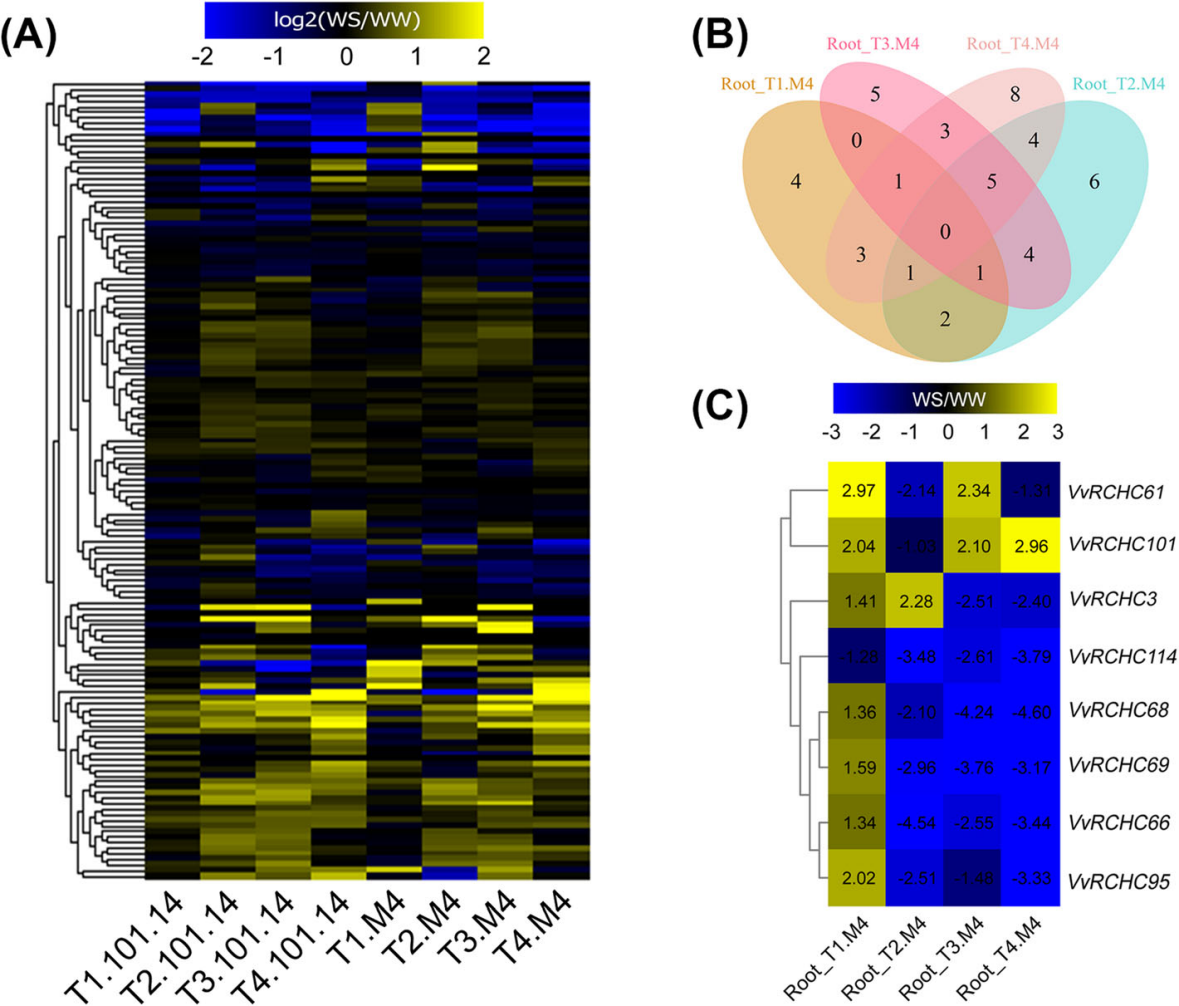
Differential heat map of *VvRCHCs* expression in plants under drought stress conditions. **a** Heat maps of two different genotypes, based on their log2(WS/WW) values from the RNA-Seq data set, under drought stress and normal conditions at times T1–T4. **b** The Venn diagram of DEGs obtained from the analysis of the expression of M4 genotype at different periods. **c** Selected eight candidate genes in genotype M4 at different stages of WS/WW heat map

**Fig. 6:**
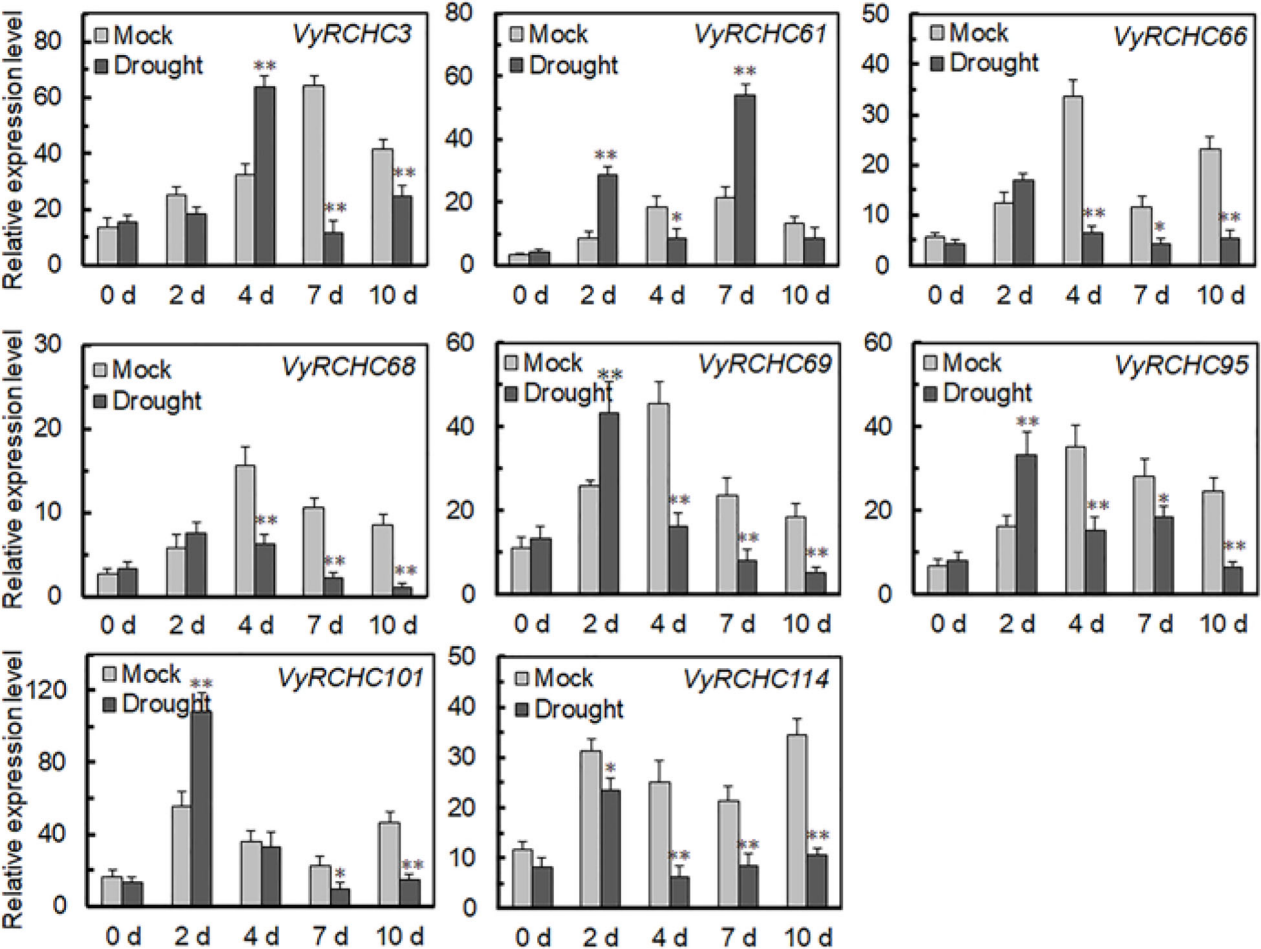
Expression of 8 candidate genes were screened in qRT-PCR for plants under drought stress and control conditions. The x-axis represents the different days during the treatment and the y-axis the relative levels of a gene’s expression. Each treatment group had three biological repeats whose averages are plotted with the standard deviation. The asterisks indicate the significant level (* *P* < 0.05, ** *P* < 0.01)

To investigate differential *VvRCHCs*’ expression between plants having contrasting drought-resistant genes (101.14 vs. M4) under drought stress and their potential functioning, the grapevine RNA-Seq transcriptome database of the published dataset was used [24]. We checked the expression of 143 *VvRCHCs*, of them, a total of 136 *VvRCHCs* expression.To understand the expression of these *VvRCHCs* under the drought treatment, we used the ratio of WS (Water Stress) to WW (Well-Watered) gene expression of the two genotypes to draw an expression heat map, expression values are reported as log_2_(WS/WW) fold change (Fig. 5a), the differential multiple matrixes of these *VvRCHCs* is recorded in Table S4. However, more than 60 % of the *VvRCHCs* in the two genotypes were highly expressed under the imposed drought. To screen out the key genes, in each time period of the treatment, the gene that conforms to |log_2_ (WS/WW)| > 1 is considered a differential gene, and the Venn diagram was made using the differentially screened genes of the drought-tolerant genotype M4 at different times (Fig. 5b). By looking at the different genes in each period, there are finally 8 genes that are different in three periods. To robustly verify the gene expression levels, the expression patterns of these 8 genes were verified by qRT-PCR (Fig. 6), whose pattern basically conformed to the trend shown in Fig. 5c. The *VyRCHC114* gene was significantly down-regulated at 2 days, with a strong downward trend of the drought treatment. The *VyRCHC66*, *VyRCHC68*, *VyRCHC69* and *VyRCHC95* genes had a similar expression trend, being slightly up-regulated at 2 days of drought, but strongly down-regulated thereafter. These results suggested eight key genes are probably involved in regulating the plant response to drought.

### Identification of E3 ubiquitin ligase activity of VyRCHC114

**Fig. 7:**
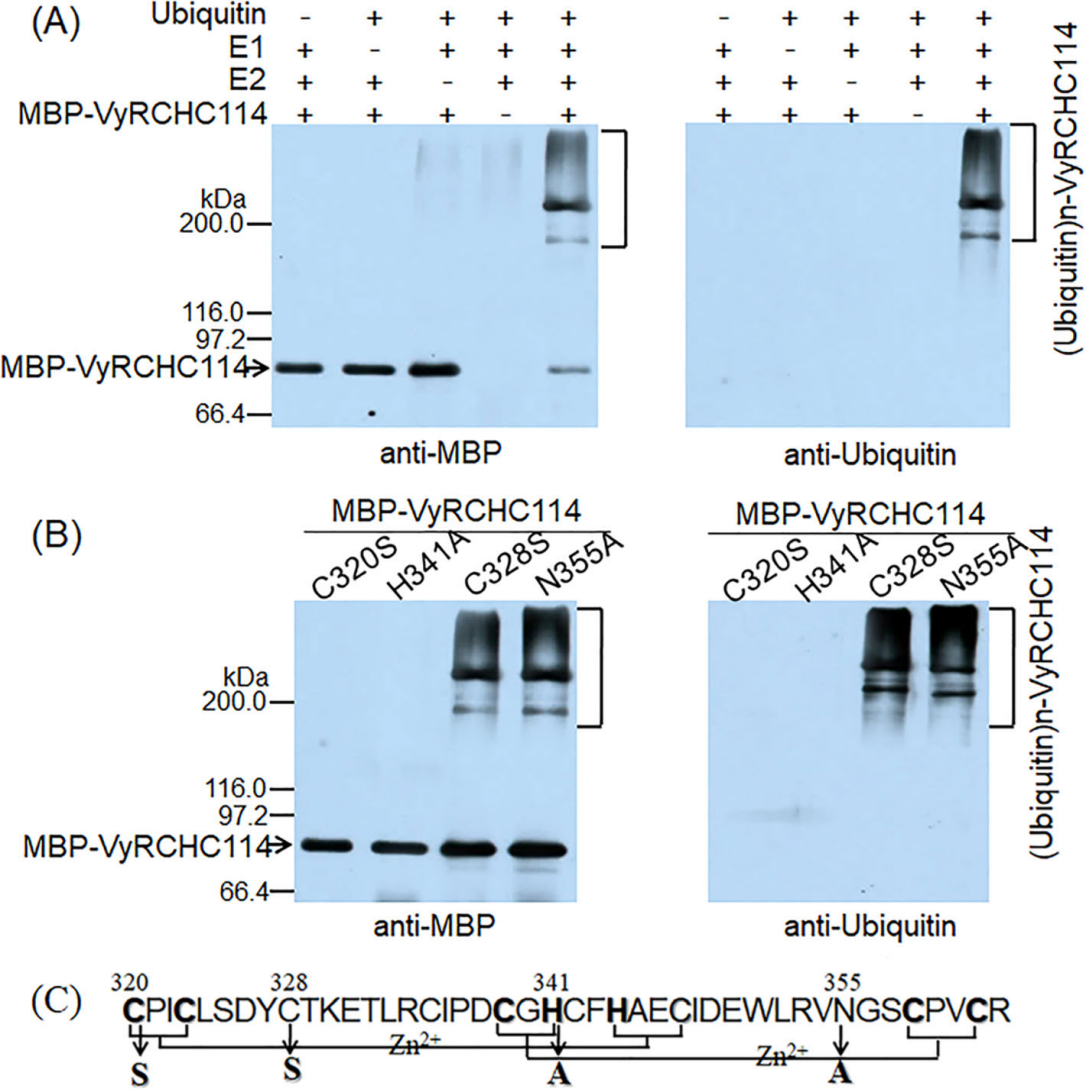
E3 ubiquitin ligase activity of VyRCHC114. **a** Determination of E3 ubiquitin ligase activity of VyRCHC114; an immunoblot analysis was performed with the ubiquitin antibody (right) and MBP antibody (left). **b** Determination of E3 ubiquitin ligase activity of VyRCHC114 mutants; an immunoblot analysis was performed with ubiquitin antibody (right) and MBP antibody (left). **c** Schematic diagram of VyRCHC114 C3H2C3 domain and putative mutation sites. C328S and N355A affect a non-conserved site of the VyRCHC114 C3H2C3 domain. Mutations in C320S and H341A affect the ubiquitin activity of *VyRCHC114*

To clarify whether VyRCHC114 has E3 ubiquitin ligase activity, we conducted an in vitro ubiquitin activity assay, achieved by using purified MBP-VyRCHC114 fusion protein mixed with ubiquitin, E1, and E2 and by western blotting with the MBP antibody. Ubiquitin molecules were detected on the fusion protein linked by MBP antibody (Fig. 7a). This same method was used to detect ubiquitin antibody tags. The VyRCHC114 protein was detected in the fusion protein linked by the ubiquitin antibody, which indicated it had E3 ligase activity.

We know that the RING-C3H2C3 type protein can form a RING structure for ubiquitin regulation, but this process depends on the interaction between the eight conserved metal ligands. To further illustrate whether and how E3 ligase activity of VyRCHC114 depends on these conserved metal ligands, as shown in Fig. 7c, we selected four different amino acid sites for mutation (two key conservative and two non-conservative metal ligand sites). Four corresponding proteins (C320S, C328S, H341A, N355A) were obtained, and their ubiquitin activity in vitro was tested by the same method. After the immuno-blotting analysis of MBP antibody and ubiquitin antibody, evidently the two mutant proteins C320S and H341A lost their E3 ubiquitin ligase activity due to mutations at key sites, but the two mutant proteins C328S and N355A maintained theirs (Fig. 7b). The unprocessed original image is in Fig. S6. These results indicated these conserved metal ligand sites are crucial factors for demonstrating the VyRCHC114 ligase activity.

### Overexpression of *VyRCHC114* enhances Arabidopsis drought tolerance

**Fig. 8:**
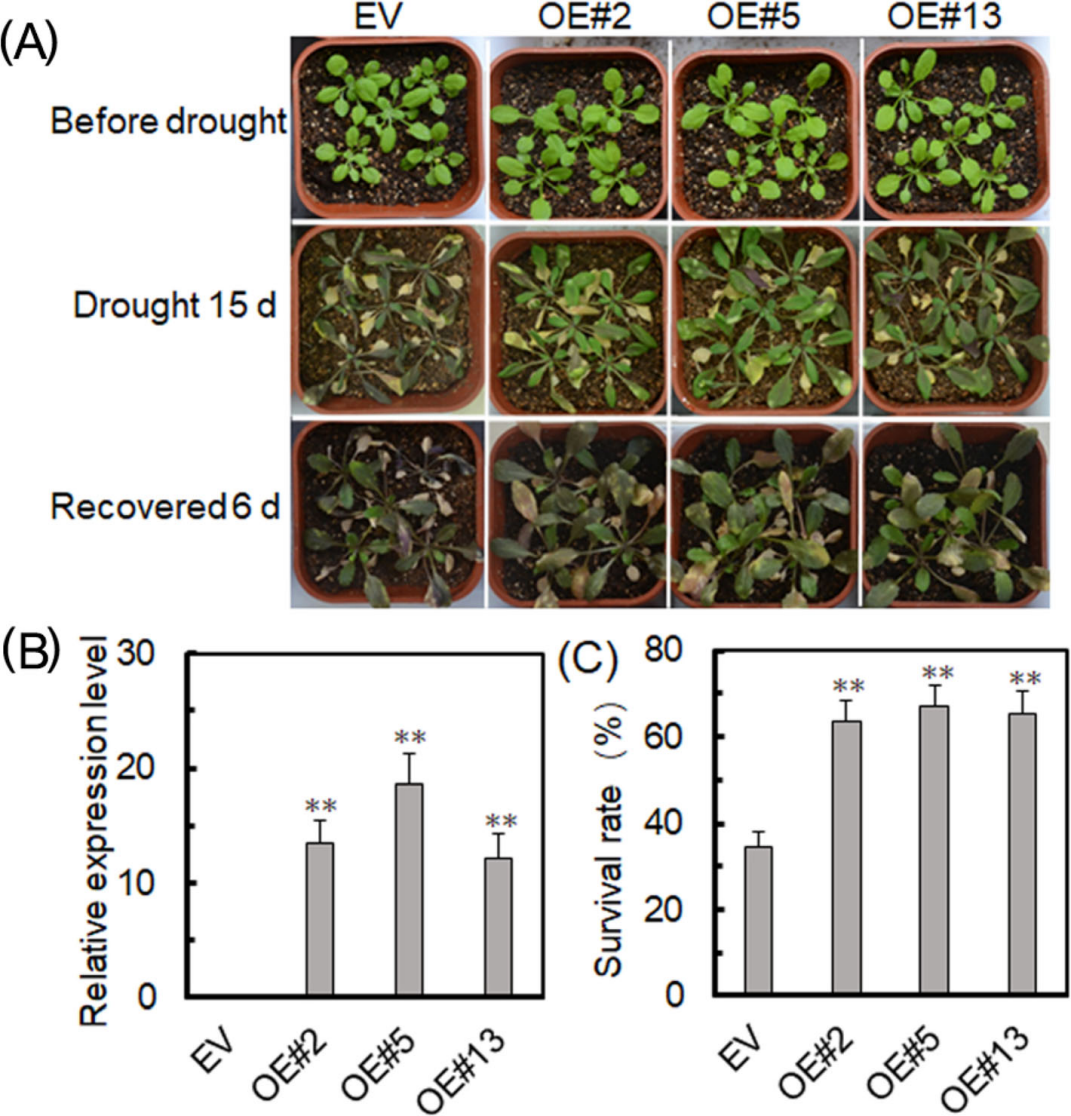
*VyRCHC114* overexpression (OE) enhances drought resistance in Arabidopsis. **a** Phenotypes of three transgenic and an EV-transformed Arabidopsis lines after 15 days of drought stress and a 6-day recovery period. **b**, **c** Relative expression levels of *VyRCHC114* and survival of transgenic and EV-transformed Arabidopsis plants. Data are the mean ± SD (standard deviation). The asterisk, (*) and (**), indicate that OEs and EV-transformed groups were significantly different at *P* < 0.05 and *P* < 0.01 (Student’s t-test)

To clarify the effects of *VyRCHC114*’s role in plant responses to drought, we selected transgenic Arabidopsis (OE #2, #5, #13) with high expression levels of the *VyRCHC114* gene for subsequent experiments (Fig. 8b). After 15 days of drought imposed upon wild plants and transgenic plants, followed by normal watering for 6 days, phenotype observations revealed that plants overexpressing *VyRCHC114* had significantly improved the drought tolerance (Fig. 8a). Further, on average, more than 70 % of the plants overexpressing *VyRCHC114* survived the drought stress, which was significantly higher than the 30 % survival rate of the EV-transformed group (Fig. 8c).

**Fig. 9:**
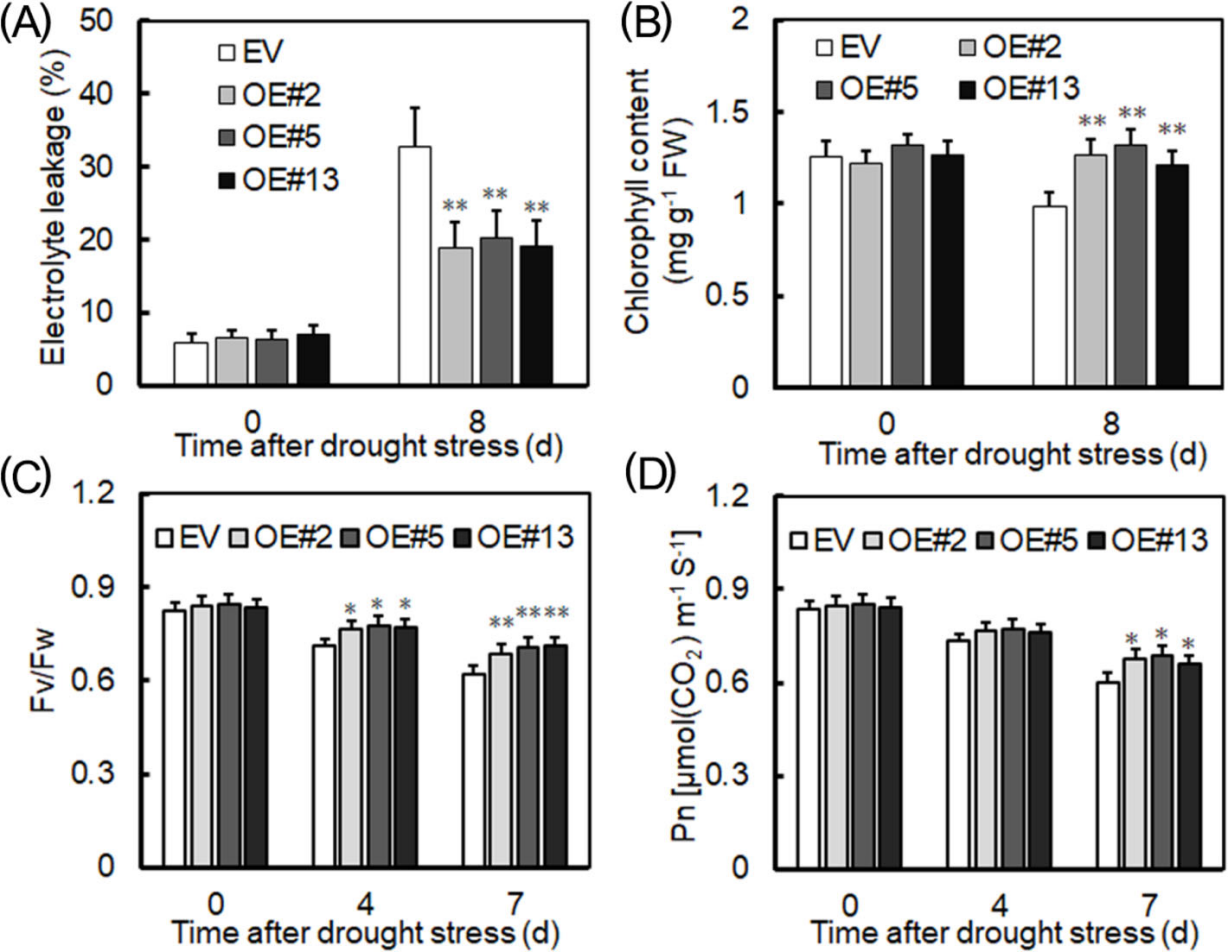
Physiological indices of the EV-transformed and overexpressing (OE) Arabidopsis plants after drought stress. **a** Electrolyte leakage, **b** chlorophyll content, **c** PSII maximal photochemical efficiency (Fv/Fm), **d** net photosynthetic rate of leaves (Pn) were evaluated. Data are the mean ± SD (standard deviation). The asterisk, (*) and (**), indicates that OEs and EV-transformed groups were significant different at *P* < 0.05 and *P* < 0.01 (Student’s t-test)

To understand the relationship between plant growth and drought resistance, electrolyte leakage rates (Fig. 9a) and chlorophyll content (Fig. 9b) were both measured. These were similar between *VyRCHC114*-overexpressed and EV-transformed plants in the non-stress treatment, but after 8 days of drought stress, the electrolyte permeability of the former was significantly lower than the latter, while the chlorophyll content was significantly higher in overexpressing than EV-transformed plants. Additionally, the changes in photosynthesis under drought stress were further analyzed by measuring potential photosynthetic efficiency (Fig. 9c) and capacity storage capacity (Fig. 9d). Each was not significantly different from EV-transformed and *VyRCHC114* overexpression plants under non-stress; however, Fv/Fm was significantly higher in the latter than the former at 4 days, and especially at 7 days, of drought stress. At 4 days, energy storage capacity of *VyRCHC114*-overexpressed plants was not significantly different from that of EV-transformed plants, but at 7 days of drought stress, that of the former exceeded the latter. Hence, these results suggest that *VyRCHC114* can enhance the drought resistance of plants by participating in the regulation of photosynthesis.

**Fig. 10:**
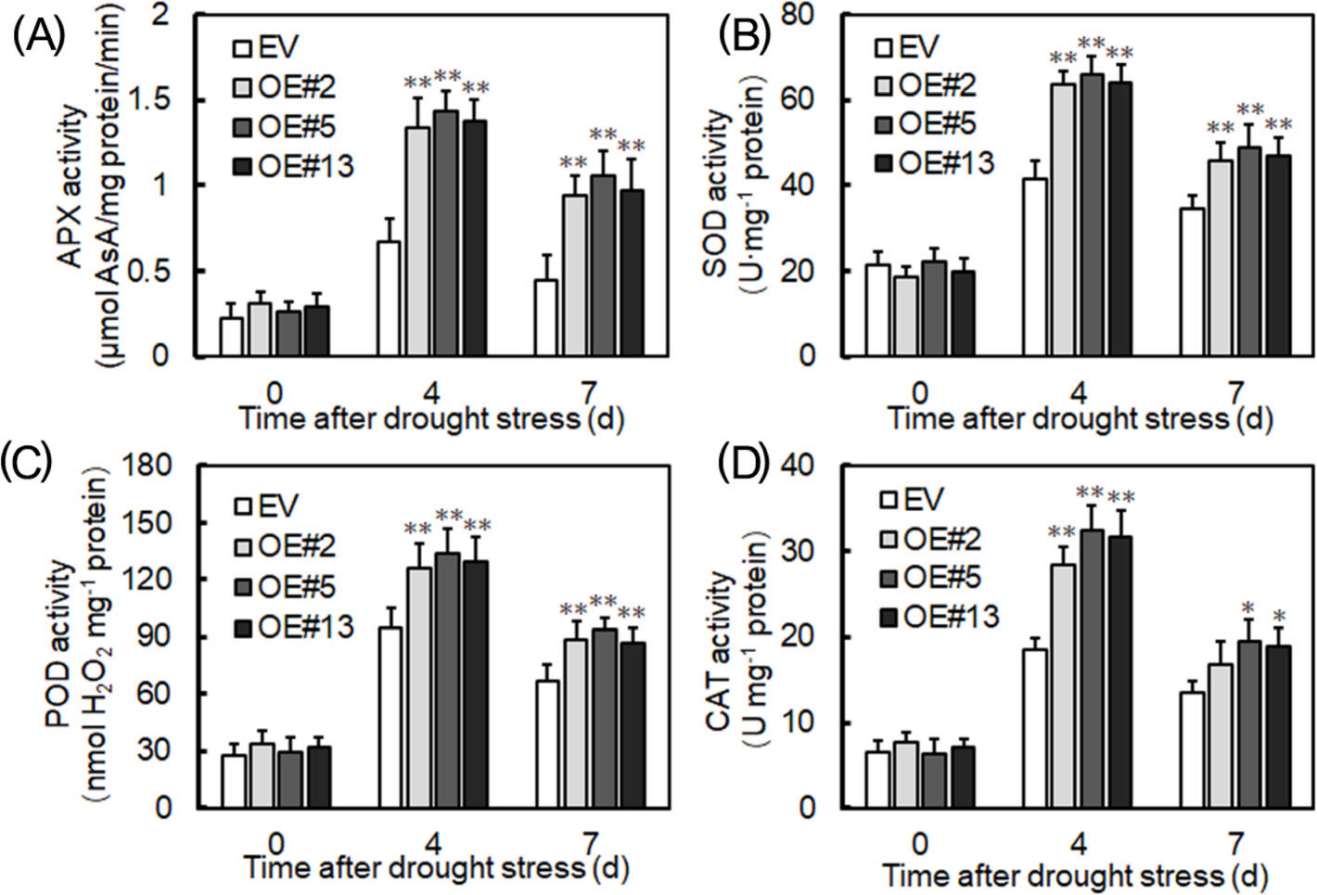
*VyRCHC114*-overexpressed and EV-transformed plants’ activity of various antioxidant enzymes in Arabidopsis. **a** Under drought stress for 0, 4, and 7 days, are ascorbate peroxidase (APX), **b** superoxide dismutase (SOD), **c** peroxidase (POD), **d** catalase (CAT) activities of overexpressed (OE) and EV-transformed plants were determined. Data are mean ± SD (standard deviation). The asterisk, (*) and (**), indicates that OEs and EV-transformed groups were significantly different at *P* < 0.05 and *P* < 0.01 (Student’s t-test)

Many studies have shown that antioxidant enzymes can influence plants’ drought tolerance. Common antioxidant enzymes are ascorbate peroxidase (APX), superoxide dismutase (SOD), peroxidase (POD), and catalase (CAT), so we examined their activity. As Fig. 10 shows, under non-stress conditions, the activity of these antioxidant enzymes was similar between the plants, whereas when drought stressed for 4 and 7 days, the activities of APX (Fig. 10a), SOD (Fig. 10b), POD (Fig. 10c) and CAT (Fig. 10d) were significantly higher in plants overexpressing *VyRCHC114* than those EV-transformed. Taken together, these data indicate *VyRCHC114* may also improve drought tolerance by elevating antioxidant enzyme activity.

**Fig. 11:**
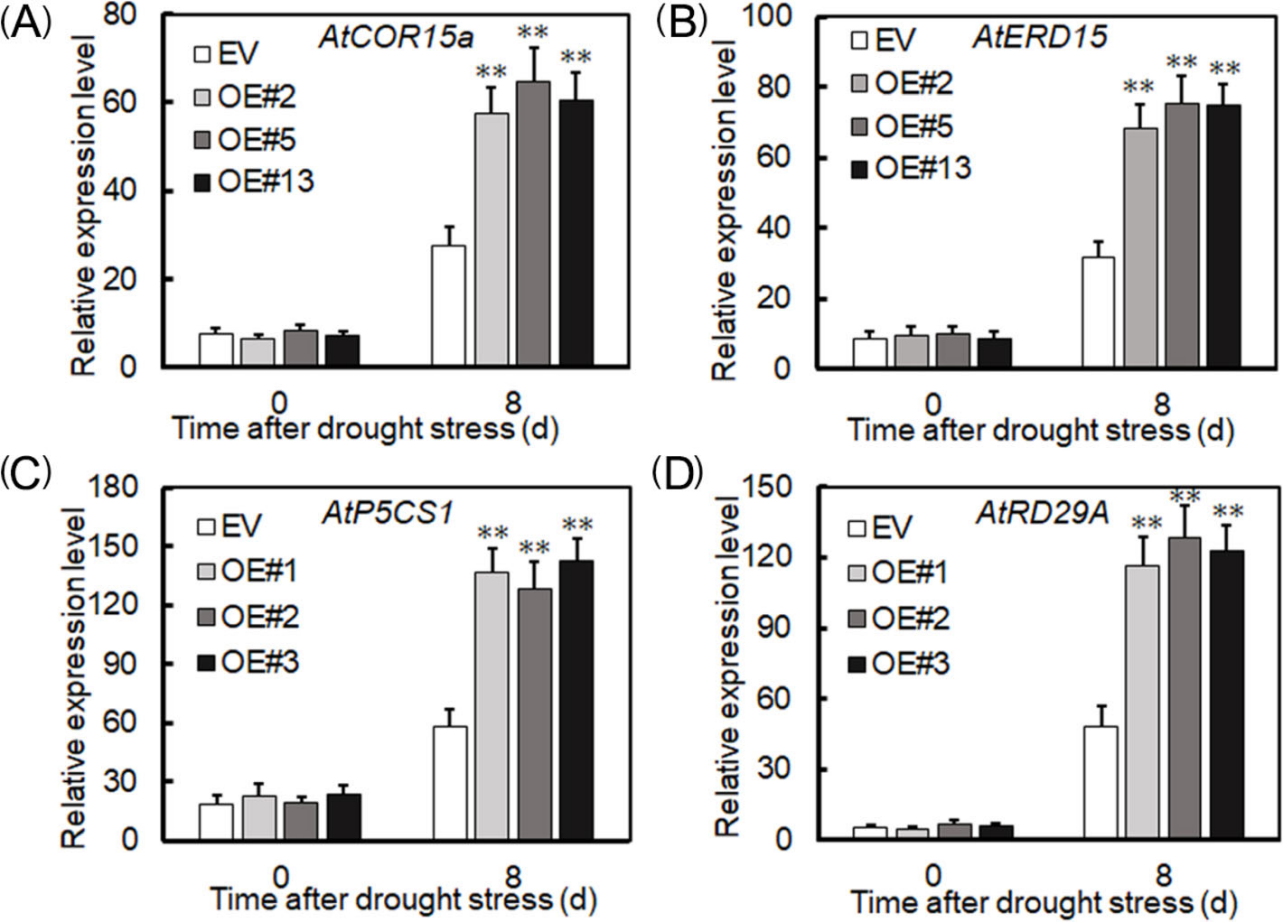
Relative expression levels of drought resistance genes in transgenic and EV-transformed Arabidopsis after drought stress. **a** AtCOR15a, **b** AtERD15, **c** AtP5CS1, **d** AtRD29A. Data are the mean ± SD (standard deviation). The asterisk, (*) and (**), indicates that overexpressed (OEs) and EV-transformed groups of plants were significantly different at *P* < 0.05 and *P* < 0.01 (Student’s t-test)

*AtCOR15a*, *AtERD15*, *AtP5CS1*, and *AtRD29A* are known to be key genes for regulating plant responses to drought stress. So we quantified their expression of imposed drought. As expected, when non-stressed, there was no significant difference between plants overexpressing *VyRCHC114* overexpression and those EV-transformed. By contrast, under drought stress, all four genes were significantly higher in *VyRCHC114*-overexpressed plants than in those EV-transformed (Fig. 11).

## Discussion

The RING C3H2C3 gene family has since been identified with many plant species [25–28]. Related studies have shown that RING genes are involved in a variety of biological processes, growth and development and hormonal responses, as well as plant responses to abiotic stresses [29]. However, for grapevine, the RING C3H2C3 gene had not yet been identified in its whole genome, with few reports available on its relevance for grapevine growth and developmental regulation or response to abiotic stress. In our study, we analyzed the whole genome of grapevine for the RING C3H2C3 gene family members. Using the criteria of whether the eight conserved metal ligands are present, a total of 143 non-redundant RING C3H2C3 genes were thus identified. Studies have shown that grapevine’s genome size is about 0.5 times that of tomato, containing 0.75 times as many genes as tomato [30, 31]. According to the known RING C3H2C3genes in tomato, the genes account for 0.58 in grapevine, which lies between the multiples of genome length and the number of genes [26].

Many RING C3H2C3 of E3 ubiquitin ligases belong to the ATL gene family [32]. According to Arabidopsis and tomato RING C3H2C3 genes, we divided grapevine’s RING C3H2C3 genes into six categories (I ~ VI) (Fig. 2). Each group has Arabidopsis or tomato in the same branch. This shows that grapevine genes have sequence similarity with *Arabidopsis thaliana* and tomato genes. Gene replication can arise from fragment replication, tandem replication, transposable events, and even whole genome replication, which not only provide the evolutionary potential for species to produce new functional traits but also are a main driving force for species differentiation [33, 34]. In the identification of gene families from many species, gene replication events have proven instrumental in their expansion [35]. Studies have shown that tandem replication often occurs in widely and fast evolving gene families, a good example being Nucleotide Binding Sites Leucine Rich Repeat (NBS-LRR) resistance families [36]. Segmental replication is more common in slow evolving gene families, like the MYB gene family [36]. There are 7 pairs of genes in *VvRCHCs* that are duplicated in tandem, and 2 pairs of genes are duplicated in segments. Tandem replication may be the cause of *VvRCHCs* expansion, like the WRKY family genes in the autopolyploid *Saccharum spontaneum* [37]. The collinearity analysis of *VvRCHCs* with Arabidopsis and tomato explains the homology relationship between grape RCHC gene and tomato is closer. In addition, 54 *VvRCHCs* were found to be homologous to genes in Arabidopsis and tomato, these genes may be preserved by the ancestors of dicots.

*Cis*-acting elements in gene promoter regions may be critical for gene regulation. Plant hormone regulation, growth and development, and stress were common upstream of different *VyRCHCs*. This situation is in fact rather common in RING genes of all species [25–28]. The ubiquitin-proteasome system has been implicated in the control of the ABA response at different points of the ABA pathway [38]. ABRE is ubiquitous in the promoter of *VvRCHCs* (Fig. S4), Ubiquitin ligase SDIR1 regulates stress-responsive abscisic acid signal by interacting with ABRE abscisic acid response element [39]. There are ABRE elements in the promoters of *VvRCHC3*,*VvRCHC16* and *VvRCHC74*, which may be induced by the regulation of abscisic acid. ABA, GA, ethylene, trauma, drought, heat stress, and pathogen response elements are present in the promoter region of *OsRING* genes of rice plants, for which pathogen infection, SA, ABA, JA, and ethephon (ET) treatments could induce target genes expression to different degrees [40]. A similar analysis of RING gene Z*mRHCP1* was recently done in maize [41]. Similarly, there are at least seven types of hormone regulatory binding elements in the promoters of *VvRCHC39*, *VvRCHC65*, *VvRCHC126* and *VvRCHC129*, they may be responding to a variety of hormone regulation.

According to the analysis of RNG-Seq data set (Fig. 5a), more than 60 % of *VyRCHCs* were significantly up-regulated or down-regulated under drought stress, indicating those genes may play a key role in how grapevine responds to drought. Studies have revealed the molecular mechanism of many circular genes involved in the drought stress. For example, in Arabidopsis, *XERICO, SDIR1, AtAIRP1, AtAIRP2, AtAIRP3* and *AtAIRP4* has been found to play a key role in the drought response of plants. In addition, E3 ubiquitin ligase *atrzf1* mutation increased the proline content of Arabidopsis and improved drought tolerance [42]. *GpDSR7* encodes an E3 ubiquitin ligase, which overexpressed in Arabidopsis increased its tolerance to drought stress [43]. In our study, focused on genes which were significantly up-regulated or down-regulated at four time periods during the drought treatment according to previous screening methods [44], as they more likely to play a key role in grapevine’s drought stress response (Fig. 5b and c). Comparing the RNA-Seq dataset with the RT-qPCR data (Fig. 6), it was found that the *VyRCHC114*continues to be downregulated. Hence, we postulated the *VyRCHC114* may possess E3 ubiquitin ligase activity and as a negative regulatory factor to respond to drought stress. Verifying this, we detected that *VyRCHC114* has E3 ubiquitin ligase activity (Fig. 7) and the *VyRCHC114* gene is overexpressed in Arabidopsis (Fig. 8). The results show the overexpression of *VyRCHC114* gave Arabidopsis drought resistance. This is not in line with expectations. Use the two reference models in Fig. S5 to explain the experimental results. Pattern 1: After VyRCHC114 degrades target protein A, protein B, which is functionally redundant with A, is strongly activated, which gives plants stronger drought resistance. Genes with redundant functions often come from the same gene family, they generally have similar conserved domains and participate in various life activities together [45]. the five *MIR172* have redundancy in the regulation of Arabidopsis meristem size, stem elongation and flowering [46]. AtUBP12 and AtUBP13 have functional redundancy in plant immunity, circadian clock, and photoperiod flowering regulation [47, 48]. Pattern 2: After VyRCHC114 degrades target protein A, protein B, which competes with A, is not inhibited, and continues to regulate downstream gene expression. This kind of competitive relationship is often accompanied by a complicated regulatory network. There are studies describing similar that *ERF4* and *MYB52* regulate downstream gene expression in an opposite manner by antagonizing each other’s DNA-binding ability through a physical interaction [49]. Fig. S5 show interesting and complex networks. The results that are not in line with expectations have prompted more attention to the target protein of VyRCHC114. The preliminary pattern diagram (Fig. S5) gives us confidence. In addition, overexpression of *VyRCHC114* caused changes in many related indexes, including antioxidant enzyme activity, photosynthesis rate, active oxygen metabolism and drought resistance gene expression. The results also indirectly prove that the targeted degradation substrate of the gene may be a key regulatory factor in the process of drought stress, and its degradation strongly activates other elements to resist drought stress, thus giving plants stronger drought resistance. It’s going to be an interesting story. In the next work, we will identify the substrate protein of *VyRCHC114*, further study its regulatory pathway, sequence the transcriptome of *Vitis yeshanensis* under drought stress, the co-expression networks under drought stress, and compare the differences of drought resistance of other cultivated species.

Drought stress greatly impacts the photosynthesis of plants, by affecting their photosynthetic rates and carbon metabolic pathways [50]. A lowered rate of photosynthesis can lead to excessive accumulation of reactive oxygen species (ROS), leading to cytotoxicity, membrane lipid peroxidation, and even cell death which can be countered by antioxidant enzymes as a form of plant defense [51, 52]. The overexpression of maize E3 ubiquitin ligase gene in transgenic tobacco can reportedly improve the drought resistance of tobacco [53]. Not only that, other abiotic stresses may also be regulated by photosynthesis, thus enabling plants to adapt to stress conditions [54]. According to our results, *VyRCHC114* overexpressing plants maintained a strong photosynthetic rate and energy storage capacity while under drought stress. The reason for this may be an increase in their chloroplast content, pointing to *VyRCHC114*’s possible involvement in the regulation of chlorophyll biosynthesis pathway as an E3 ubiquitin ligase. Nonetheless, we also examined the expression of genes known to play a major role in drought stress responses to plants [55–57]. Our results revealed that the expression levels of these genes were significantly higher in *VyRCHC114*-overexpressed than EV-transformed Arabidopsis *plants*. Moreover, antioxidant system may be involved in plant abiotic stress tolerance mediated by the E3 ubiquitin ligase. Here, we provide physiological evidence that *VyRCHC114* heterologous expression enhances drought resistance by increasing the activity of antioxidant enzymes, which can scavenge for and eliminate ROS to indirectly reduce membrane damage.

## Conclusions

*VyRCHC* may act as an E3 ligase to mediate substrate degradation through the ubiquitin proteasome mechanism. This interaction may cause the target protein to be labeled by ubiquitin signaling, which leads to proteasome degradation. Since *VyRCHC114* likely represents a new class of positive /negative regulatory factors of the drought signal pathway, however, positive/negative depends on the regulatory characteristics of the target protein, but we think the degraded protein is a positive regulator of drought signaling, so that more of this substance may activate drought signaling. Therefore, *VyRCHC114* may improve the water retention ability and antioxidant defense of plants by regulating their chlorophyll content and antioxidant system, thus participating in drought stress response. So far, however, the target protein of the plant *VyRCHC114* gene has not been determined, nor is the mechanism of augmented SOD, POD, APX and CAT activities clearly understood. In future work, we will focus on the identification of *VyRCHC114* target proteins under drought stress and activation mechanisms of the antioxidant system in *VyRCHC114*-transgenic plants.

## Methods

### Plant materials

The grapevine variety *Vitis yeshanensis* was sampled from the field, located in the grape germplasm resource garden of Northwest A&F University. Annual plants are selected for treatment, and the treatment method is the same [58]. Root samples were taken at 0d, 2d, 4d, 6d, 8d and 10d respectively for subsequent experiments. Transgenic and wild type (WT) plants of *Arabidopsis thaliana* ecotype Columbia (Col-0) plants were grown in vermiculite: perlite (1:1, v/v) mix in plastic pots in a growth chamber. Arabidopsis plants were grown in a soil mix of peat moss, perlite, and vermiculite (3:1:1, v/v/v) under a 12-h/12-h day/night cycle at 25℃ with 60 % relative humidity. For the drought stress treatment, plants were transformed with an empty vector (EV) or to overexpress *VyRCHC114* (OE#2, OE#5, or OE#13 lines) [59], all of which were grown on individual MS medium plates for 7 days before transplantation into soil, there were 5 strains in the control and three transgenic lines. This experiment mainly followed previous research methods, albeit slightly modified [60]. After 3 weeks, these plants received a 12-day drought stress treatment (no water provided), after which they were re-watered and their survival recorded 6 days later. All experiments were repeated three times.

### Identification of RING-type C3H2C3 genes in the grapevine genome

To identify the C3H2H3 type of RING, the most recent grapevine genome file in the Ensembl Plants Database (http://plants.ensembl.org/index.html) was downloaded and used. The grapevine RING C3H2C3 candidates were identified based on the HMM profiles (PF13639 and PF12678) with an e-value cutoff of 0.01. The screened proteins screened were given to Pfam (http://pfam.xfam.org/search/) and SMART ( ttp://smart.embl-heidelberg.de/), e value less than 0.01. According to the results of PFAM, SMART database protein domain identification, extract RCHC conservative domain sequences in the VyRCHC protein sequence, and use CLUSTALX 2.0 to perform multi-sequence alignment, see if conserved Cys-Cys-Cys-His-His-Cys-Cys-Cys. Finally, 143 proteins that ultimately meet the conditions. The physicochemical properties of each RING-type C3H2C3 protein were predicted using the Protparam online tool (https://web.expasy.org/protparam/). The 143 *VvRCHCs* were named according to their positional information on the chromosomes.

### Bioinformatics analysis of *VyRCHCs* family

CLUSTALX 2.0 software was used to perform a multiple-sequence alignment of the 143 grapevine genes and the 18 tomato and 19 Arabidopsis RCHC protein sequences; it was also used to manually remove any untrusted gaps at both sequence ends. A phylogenetic tree was generated in MEGA 7.0 using the ML (maximum likelihood) method and bootstrapping with n = 1000 replicates, with all other settings set to their default values; the online EVOLVIEW (https://www.evolgenius.info/evolview/#login/) tool carried out the tree’s visualization. The online program Gene Structure Display Server 2.0 (http://gsds.cbi.pku.edu.cn/) was used to identify the genetic structure the *VvRCHCs*. Using the MEME online program (http://MEME.nbcr.net/meme/introduction.html), the VvRCHC protein sequences could be analyzed under these parameters: an optimal motif width of 6 ~ 35 and a maximum number of motifs of 12. According to the annotated positions in grapevine genome data, the 143 grapevine *VvRCHCs* were located on 20 chromosomes. By referring to previous studies, BLASTN was used to compare the CDS sequences of *VvRCHCs* in grapevine and tomato (e-value = 1 × 10^− 10^, homology > 75 %). The tandem repeat gene pairs and segment repeat gene pairs of *VyRCHCs* were also identified [61, 62]. Further, the Ka/Ks ratio between repetitive genes pairs can be used to infer the selection pressure in the process of genome evolution. Next, the MCScanX program (e-value: 1 × 10^− 10^, num alignments: 5) was used to detect the collinear region between *VvRCHCs* in grapevine and tomato/Arabidopsis; any collinear gene pair of *VvRCHCs* was marked with red and green lines. The cis-elements were identified from the upstream 2-kb promoter sequences of the *VvRCHCs* after submitting them to PlantCARE (http://bioinformatics.psb.ugent.be/webtools/plantcare/html) [63], to obtain their image display, the resulting XML file was uploaded to TBtools [64].

### Expression analysis of *VyRCHCs* in grapevine under drought stres

To analyze the grapevine *RCHC* genes’ expression levels under drought stress, we obtained from the NCBI database (registration number: SRA110531) two different drought resistance genes (101.14 and M4) which were compared under two different treatments WS(Water Stress) and WW (Well-Watered) in roots and in different periods (T1–T4: 2d、4d、7d、10d) RNA-Seq data set [24]. Based on the expression values of RING C3H2C3 in the roots of the two genotypes, we calculated the log_2_(WS/WW) values (fold-change) in each time period (Table S4). The R package ‘pheatmap’ was used to produce a heatmap for this data.

### RNA extraction and quantitative real-time PCR (qRT-PCR)

The qRT-PCR primers were designed using Primer Premier software (version 5.0). The RNA from Arabidopsis and grapevine (*Vitis yeshanensis*) leaves was extracted using the Spectrum Plant Total RNA Kit (Sigma-Aldrich, Beijing, China), after which reverse transcription of RNA into cDNA was done using the Prime Script RT Reagent Kit (Takara, Dalian, China). The qRT-PCR was performed in an IQ^5^ real-time PCR detection system (Bio-Rad Laboratories, Hercules, CA, USA) with SYBR Premium EX Taq II (Takara, Dalian, China). The reaction volume was 25 µl. The relative expression level corresponding to *β-TUB4* and *ubiquitin1* was calculated by using the 2^−ΔΔCt^ method [65]; each reaction was prepared in triplicate and repeated three times. Primer sequence information in Table S1.

### E3 ubiquitin ligase activity assay

The open reading frame (ORF) of VyRCHC114 and the different site mutants C320S, H341A, C328S, and N355A were separately cloned into the SalI/KpnI site of the pMAL-c5X vector (New England Biolabs UK Ltd, Hitchin, UK). According to the manufacturer’s instructions, the pMAL protein fusion and purification system (New England Biolabs) was used to purify the fusion protein. Ubiquitination activity was then measured that according to the method described above [66], albeit with the following modifications made: 250 ng of purified E3 (MBP-*VyRCHC114*, C320S, H341A, C328S, and N355A) in the ubiquitination buffer (50 mM Tris-HCl (pH 7.5), while the other reagents and steps used were the same. Primer sequence information in Table S1.

### Physiological analysis of drought stress response of transgenic Arabidopsis

To determine the water loss rate, 10 leaves were detached from 3-week-old transgenic and WT plants and immediately weighed. The samples were then placed on dry filter paper at a relative humidity of 40–45 % at room temperature and weighed over a time course. Leaves were sampled after dehydration to detect cell death, electrolyte leakage, malondialdehyde, antioxidant enzyme activity. The leaves collected before dehydration were used as a negative control.

For chlorophyll content measurements, approximately 0.05 g of fresh leaf material was placed in 5 ml of 96 % ethanol and incubated at 4◦C in the dark overnight. The absorbance of the extracted pigments was measured at 665 and 649 nm using a spectrophotometer (Hitachi Limited, Tokyo, Japan) and the chlorophyll content was calculated as previously described [58].

Relative electrolyte leakage was measured as previously described [67], as was MDA content [66]. In addition, superoxide dismutase (SOD), peroxidase (POD), catalase (CAT), ascorbate peroxidase (APX) enzyme activities were extracted from 0.5 g leaves from abiotic stress treated plants as well as control plants, and measured as described by [68].

### Statistical analysis

All the above experiments by SPSS software (version 21.0) were employed to analyze the statistically significant differences of the gene expression levels by ANOVA with Duncan’s multiple range test. All experiments were repeated three times as independent analyses.

## Supplementary Information

**Additional file 1: Figure S1. **Schematic diagram of C3H2C3 conserved sequence alignment of VvRCHCs. Cys(C) and His(H) amino acids were added on a blue and pink background. The C3H2C3 conserved amino acid sequence length of these genes is shown later in the sequence.

**Additional file 2: Figure S2. **The original tree of Fig. 2.

**Additional file 3: Figure S3. **Number of introns in *VvRCHCs*.

**Additional file 4: Figure S4. **Analysis of Cis-acting elements in the *VvRCHCs*. **a** A list of *VvRCHCs* to facilitate correspondence. **b** Color-coded numbers of cis-acting elements of the three major types of *VvRCHCs*promoters. **c** Different types of *cis-acting*elements are represented by different colored squares and their position on each *VvRCHC* gene promoter.

**Additional file 5: Figure S5. **Two model diagrams (VyRCHC114 is involved in drought resistance).

**Additional file 6: Figure S6. **VyRCHC114 in vitro ubiquitin gel imprinting (uncut). Corresponding to the original image of gel imprinting in Fig.7A and B.

**Additional file 7: Table S1. **The sequences of the primers used in these experiments.

**Additional file 8: Table S2. **The distance between conserved metal ligands in the C3H2H3 domain of 143 *VvRCHCs*.

**Additional file 9: Table S3. **Functions of the cis-acting elements that found in the promoter region of each of *VyRCHCs*.

**Additional file 10: Table S4. **The number of *VvRCHCs* expressed in the roots of two different genotypes was expressed as log_2_(WS/WW), and T1-T4 represented different drought stress time.

## Data Availability

All data generated and analyzed during this study are included in this published article. To identify the C3H2H3 type of RING, the most recent grapevine genome file in the Ensembl Plants Database ( http://plants.ensembl.org/index.html ) was downloaded and used. Expression data of C3H2H3 type of RING genes in grapevine used in this study can be accessed via the NCBI SRA database with accession numbers of SRA110531. Figure S1 Schematic diagram of C3H2C3 conserved protein sequence alignment of VvRCHCs. Figure S2 The original tree of Fig. 2. Figure S3 Number of introns in *VvRCHCs*. Figure S4 Analysis of *cis*-acting elements in the *VvRCHCs*. Figure S5. Two model diagrams (VyRCHC114 is involved in drought resistance). Figure S6 VyRCHC114 in vitro ubiquitin gel imprinting (uncut). Table S1 The sequences of the primers used in these experiments. Table S2 The distance between conserved metal ligands in the C3H2H3 domain of 143 VvRCHCs. Table S3 Functions of the *ci*s-acting elements that found in the promoter region of each of *VvRCHCs*. Table S4 Differential multiple expression matrix of *VvRCHCs*.

## Abbreviations

RCHCs: RING C3H2C3-type genes
DEGs: Differentially expressed genes
qRT-PCR: Quantitative real-time reverse transcription PCR
RNA-seq: RNA-sequencing
WS: Water stress
WW: Well-watered
ML: Maximum likelihood
WT: Wild type
HMM: Hidden Markov Model
