# Supplementary material for: Identification of C3H2C3-type RING E3 ubiquitin ligase in grapevine and characterization of drought resistance function of VyRCHC114
Source: BMC Plant Biol. 2021 Sep 17;21:422. doi: 10.1186/s12870-021-03162-8 (PMC8447581; doi:10.1186/s12870-021-03162-8)
Supplement: Supplementary file 1 — Additional file 1: Figure S1. Schematic diagram of C3H2C3 conserved sequence alignment of VvRCHCs. Cys(C) and His(H) amino acids were added on a blue and pink background. The C3H2C3 conserved amino acid sequence length of these genes is shown later in the sequence. [file 12870_2021_3162_MOESM1_ESM.docx]

**Supplementary Figure**


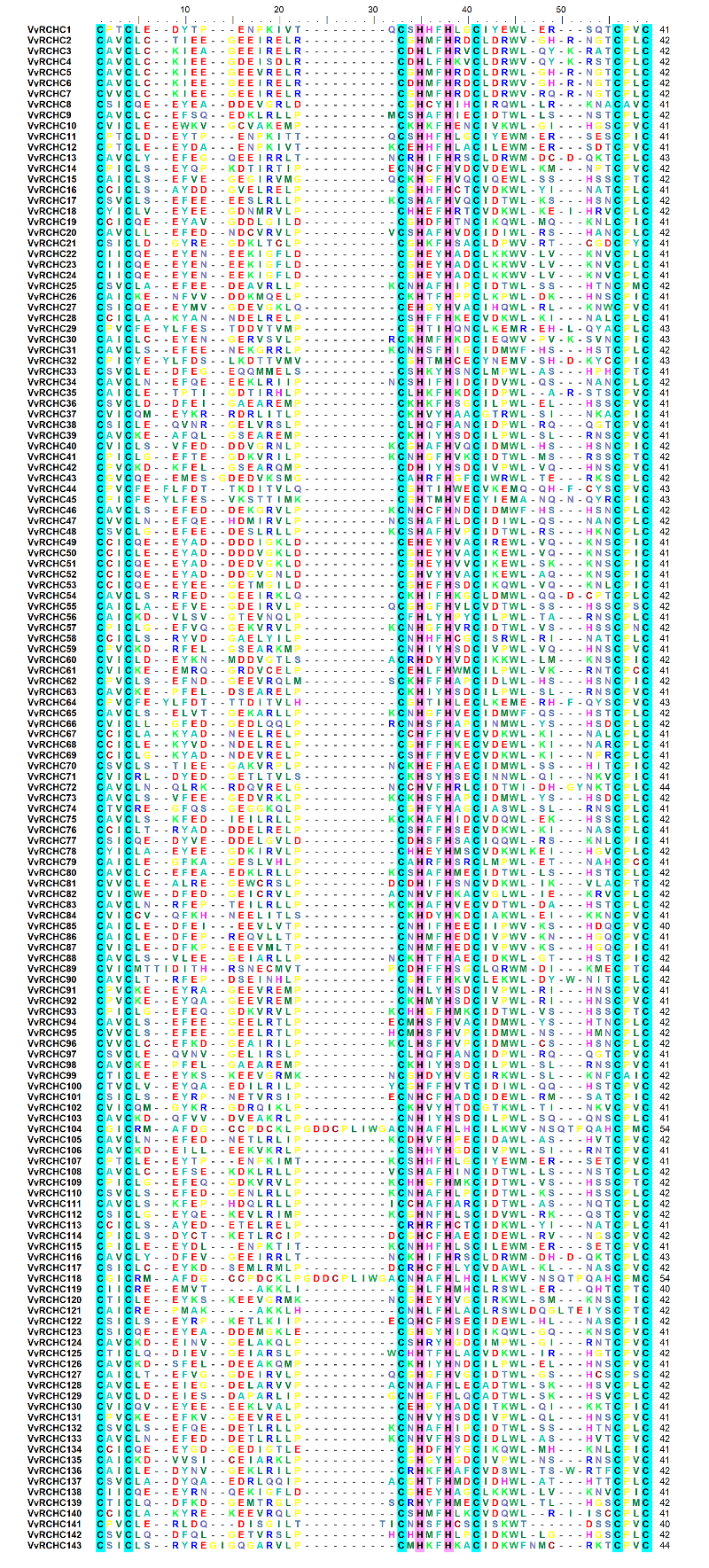


**Supplementary Figure 1** Schematic diagram of C3H2C3 conserved sequence alignment of *VvRCHCs*. Cys(C) and His(H) amino acids were added on a blue and pink background. The C3H2C3 conserved amino acid sequence length of these genes is shown later in the sequence.
