# Supplementary figures and images for: Identification of C3H2C3-type RING E3 ubiquitin ligase in grapevine and characterization of drought resistance function of VyRCHC114
Source: BMC Plant Biol. 2021 Sep 17;21:422. doi: 10.1186/s12870-021-03162-8 (PMC8447581; doi:10.1186/s12870-021-03162-8)

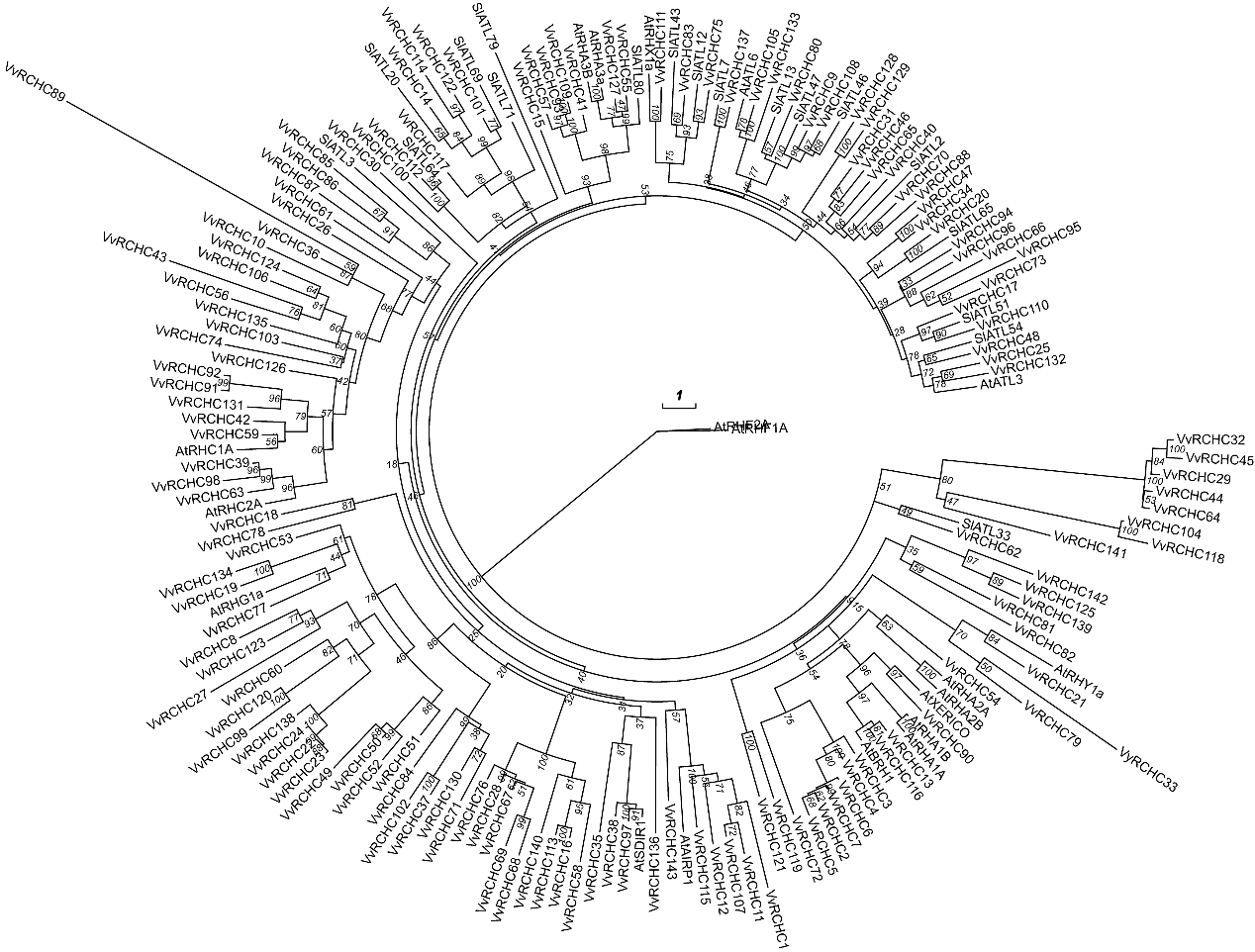


**Supplementary Figure 2. The original tree of Fig. 2.**

Supplement: Supplementary file 2 — Additional file 2: Figure S2. The original tree of Fig. 2. [file 12870_2021_3162_MOESM2_ESM.docx]

**
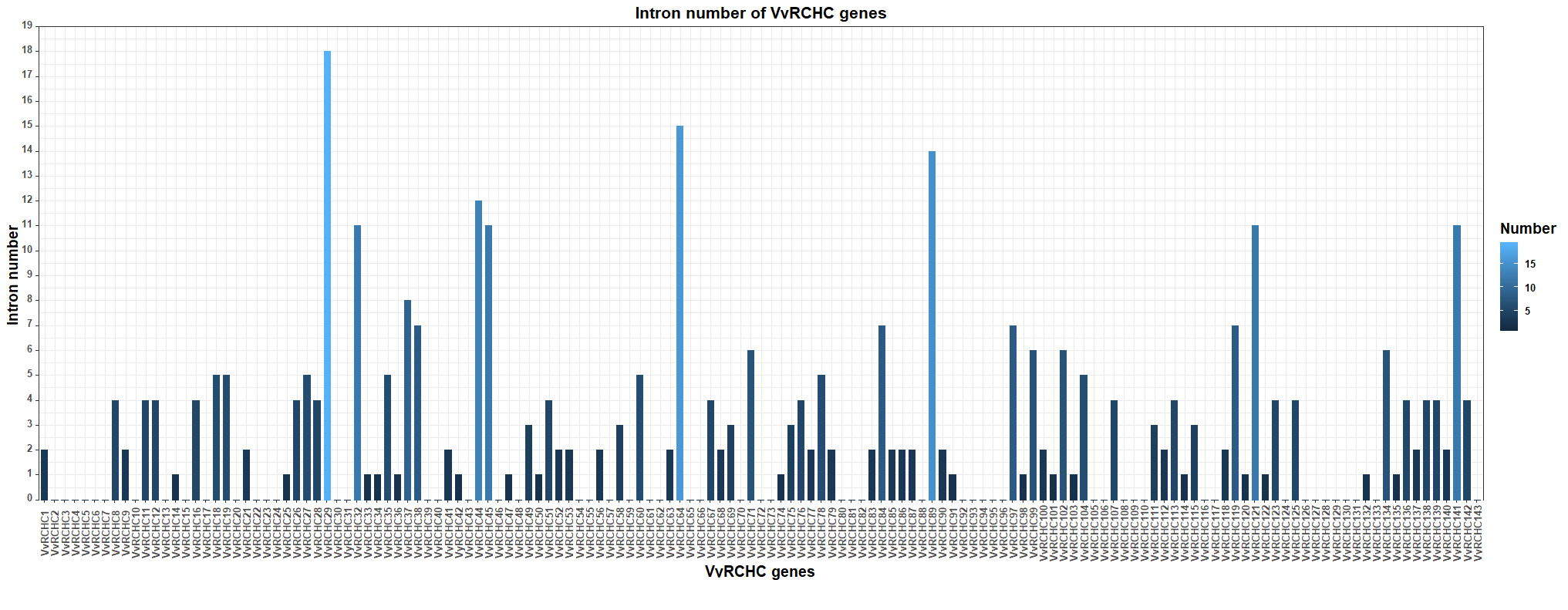
**

**Supplementary Figure 3 Number of introns in *VvRCHCs*.**

Supplement: Supplementary file 3 — Additional file 3: Figure S3. Number of introns in VvRCHCs. [file 12870_2021_3162_MOESM3_ESM.docx]

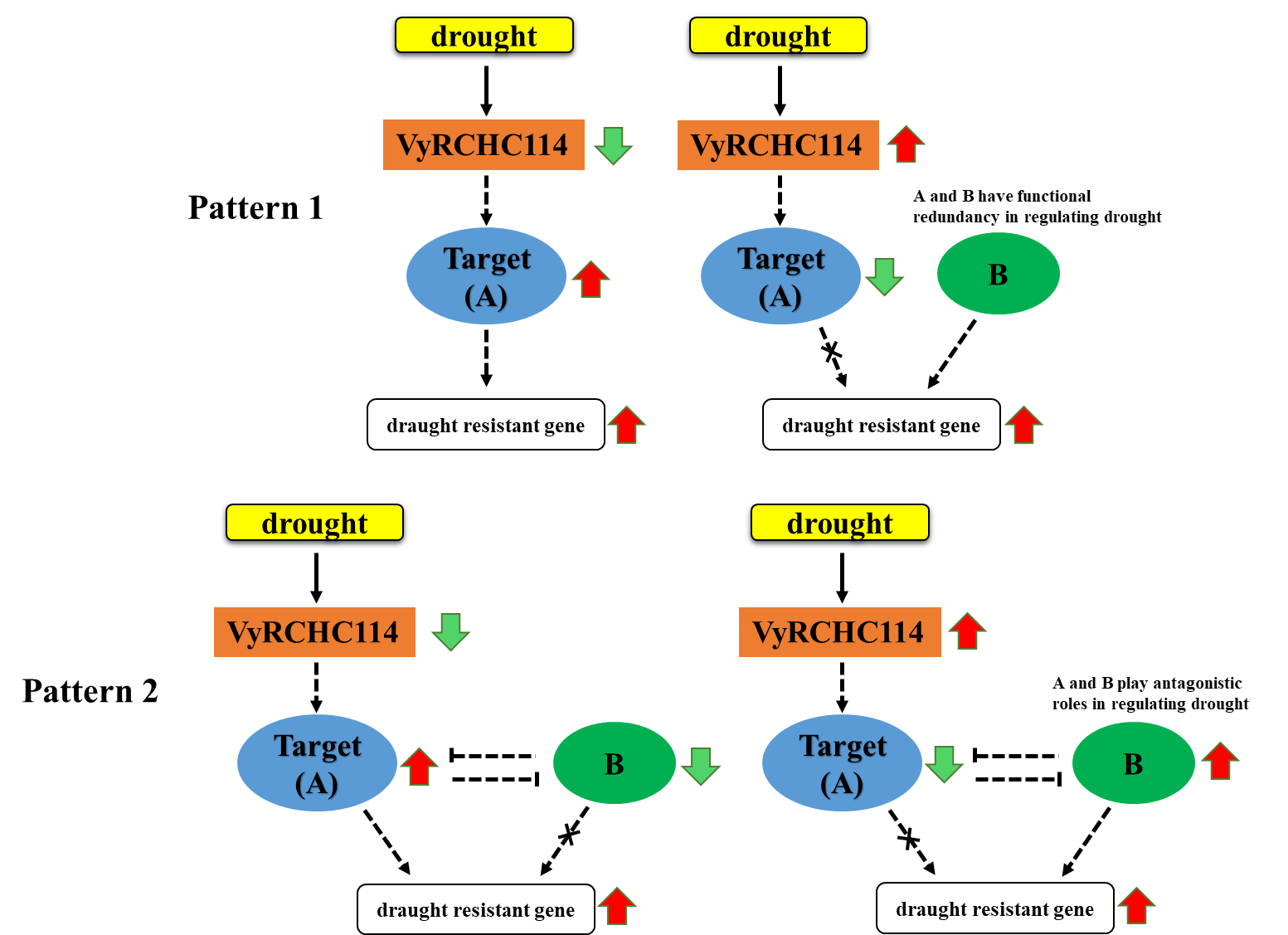


**Supplementary** **Figure S5. Two model diagrams (VyRCHC114 is involved in drought resistance).**

Supplement: Supplementary file 5 — Additional file 5: Figure S5. Two model diagrams (VyRCHC114 is involved in drought resistance). [file 12870_2021_3162_MOESM5_ESM.docx]
