# Supplementary material for: Identification of C3H2C3-type RING E3 ubiquitin ligase in grapevine and characterization of drought resistance function of VyRCHC114
Source: BMC Plant Biol. 2021 Sep 17;21:422. doi: 10.1186/s12870-021-03162-8 (PMC8447581; doi:10.1186/s12870-021-03162-8)
Supplement: Supplementary file 4 — Additional file 4: Figure S4. Analysis of Cis-acting elements in the VvRCHCs. a A list of VvRCHCs to facilitate correspondence. b Color-coded numbers of cis-acting elements of the three major types of VvRCHCspromoters. c Different types of cis-actingelements are represented by different colored squares and their position on each VvRCHC gene promoter. [file 12870_2021_3162_MOESM4_ESM.docx]

.
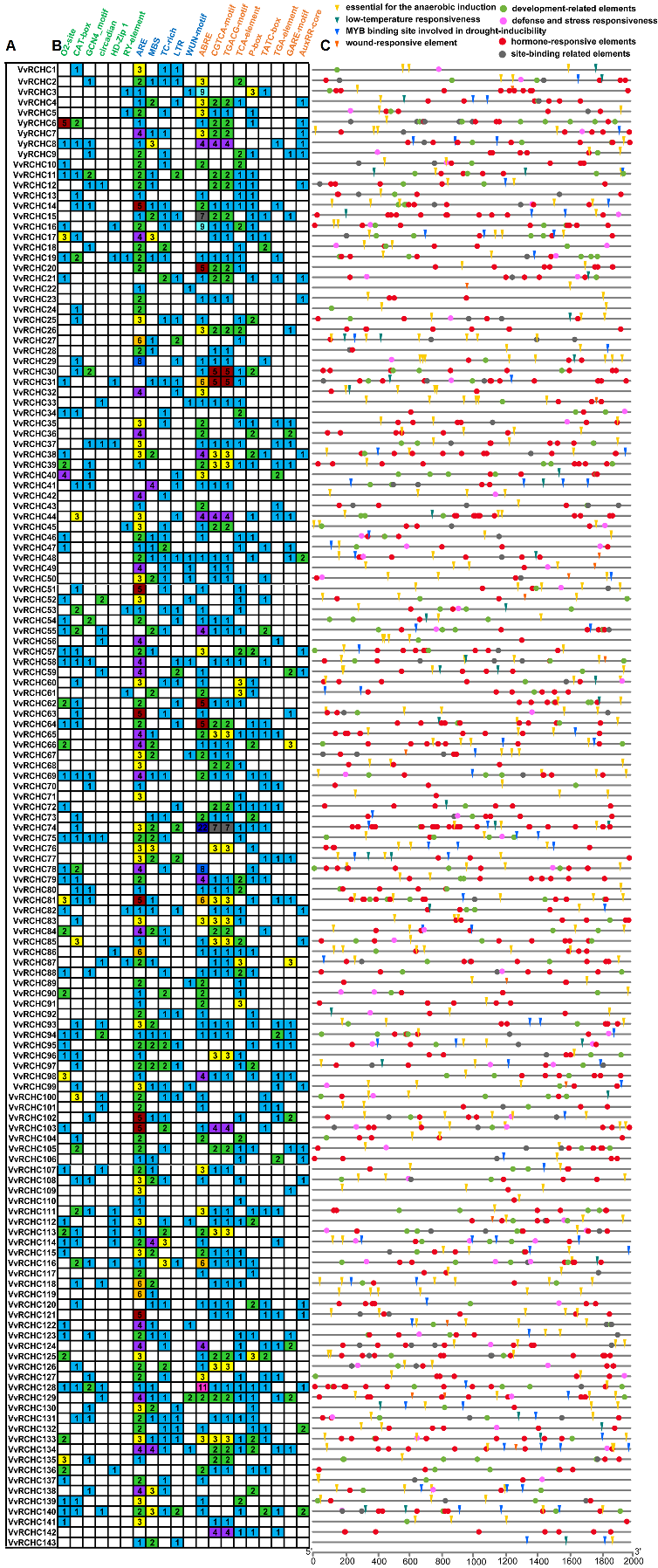


**Supplementary** **Figure 4. Analysis of cis-acting elements in the *VvRCHCs*.**

**a** A list of VvRCHCs to facilitate correspondence. **b** Color-coded numbers of cis-acting elements of the three major types of *VvRCHCs* promoters. **c** Different types of cis-acting elements are represented by different colored squares and their position on each *VvRCHC* gene promoter.
