# Supplementary material for: Identification of C3H2C3-type RING E3 ubiquitin ligase in grapevine and characterization of drought resistance function of VyRCHC114
Source: BMC Plant Biol. 2021 Sep 17;21:422. doi: 10.1186/s12870-021-03162-8 (PMC8447581; doi:10.1186/s12870-021-03162-8)
Supplement: Supplementary file 6 — Additional file 6: Figure S6. VyRCHC114 in vitro ubiquitin gel imprinting (uncut). Corresponding to the original image of gel imprinting in Fig.7A and B. [file 12870_2021_3162_MOESM6_ESM.docx]

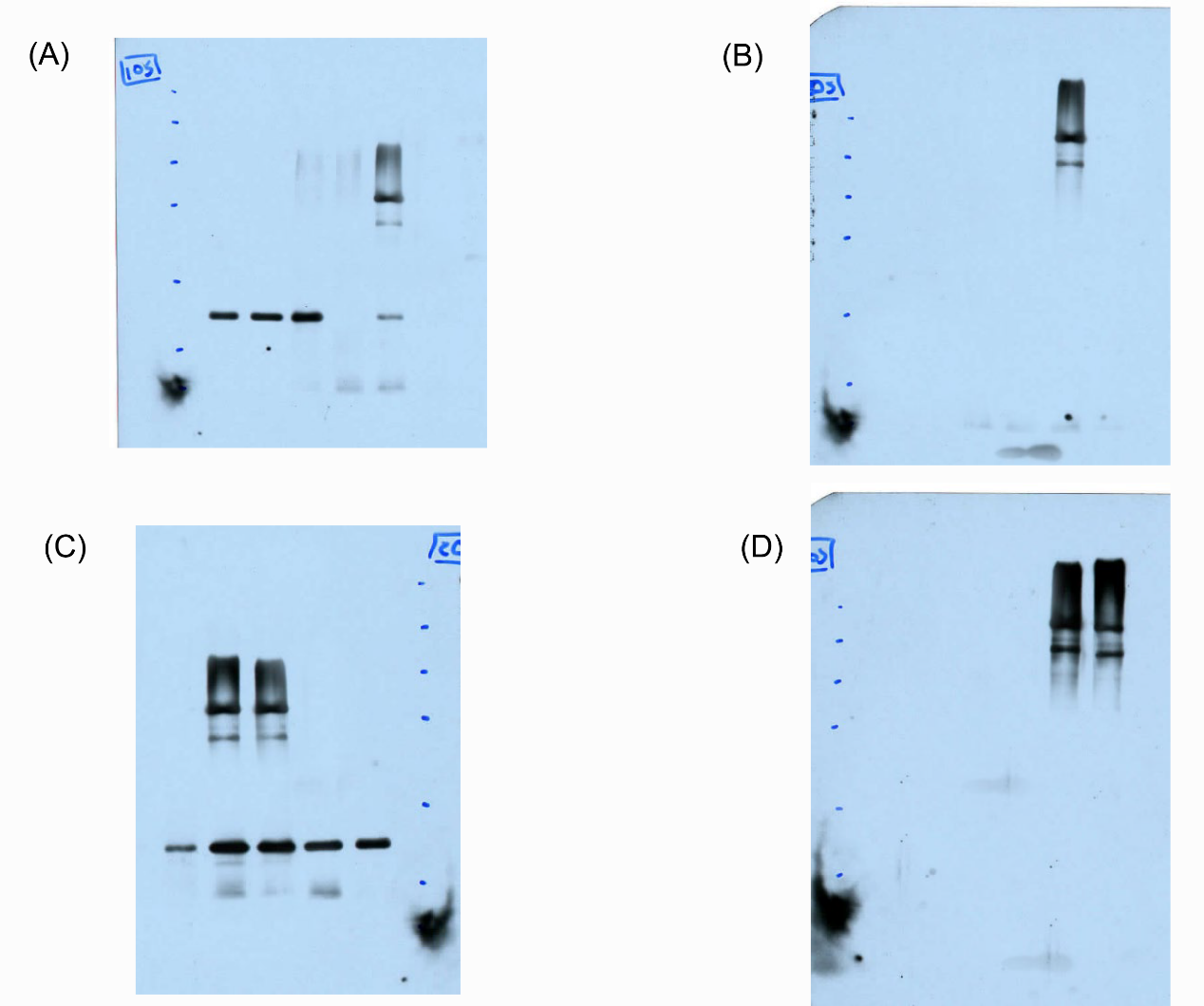


**Supplementary** **Figure 6: *VyRCHC114* in vitro ubiquitin gel imprinting (uncut).**

Corresponding to the original image of gel imprinting in Fig.7 A B C D.
